# The pathophysiology and management of depression in cardiac surgery patients

**DOI:** 10.3389/fpsyt.2023.1195028

**Published:** 2023-10-20

**Authors:** Tony Vu, Julian A. Smith

**Affiliations:** ^1^Department of Cardiothoracic Surgery, The Alfred Hospital, Melbourne, VIC, Australia; ^2^Department of Surgery, School of Clinical Sciences at Monash Health, Monash University, Melbourne, VIC, Australia; ^3^Department of Cardiothoracic Surgery, Monash Health, Melbourne, VIC, Australia

**Keywords:** postoperative depression, cardiac surgery, autonomic nervous system dysregulation, hypothalamic pituitary adrenal axis dysregulation, inflammation, cognitive behavioral therapy, antidepressant, statins

## Abstract

**Background:**

Depression is common in the cardiac surgery population. This contemporary narrative review aims to explore the main pathophysiological disturbances underpinning depression specifically within the cardiac surgery population. The common non-pharmacological and pharmacological management strategies used to manage depression within the cardiac surgery patient population are also explored.

**Methods:**

A total of 1291 articles were identified through Ovid Medline and Embase. The findings from 39 studies were included for qualitative analysis in this narrative review.

**Results:**

Depression is associated with several pathophysiological and behavioral factors which increase the likelihood of developing coronary heart disease which may ultimately require surgical intervention. The main pathophysiological factors contributing to depression are well characterized and include autonomic nervous system dysregulation, excessive inflammation and disruption of the hypothalamic–pituitary–adrenal axis. There are also several behavioral factors in depressed patients associated with the development of coronary heart disease including poor diet, insufficient exercise, poor compliance with medications and reduced adherence to cardiac rehabilitation. The common preventative and management modalities used for depression following cardiac surgery include preoperative and peri-operative education, cardiac rehabilitation, cognitive behavioral therapy, religion/prayer/spirituality, biobehavioral feedback, anti-depressant medications, and statins.

**Conclusion:**

This contemporary review explores the pathophysiological mechanisms leading to depression following cardiac surgery and the current management modalities. Further studies on the preventative and management strategies for postoperative depression in the cardiac surgery patient population are warranted.

## 1. Introduction

Cardiovascular disease (CVD) is a leading cause of mortality, accounting for over 18 million deaths globally in 2019 (1). As a management modality, over 2 million cardiac surgeries are performed globally *per annum* (2). Common examples of cardiac surgery include coronary artery bypass graft (CABG), valve replacements and heart transplantation. Given the aging population, patients who undergo cardiac surgery are more likely to be older and possess significant medical comorbidities such as hypertension and diabetes mellitus (3). Cardiac surgery intends to offer definitive management for persistent cardiovascular disease which is refractory to medical management. Successful cardiac surgery significantly improves quality of life, which in turn improves psychological outcomes. Depression is a mood disorder with a lifetime prevalence between 2 and 21% (4). Within the cardiac surgery population, the prevalence of depression is significantly higher. The prevalence of pre-operative depression ranges from 20 to 47% while postoperative depression affects 23–61% of patients following cardiac surgery (5–11). The disparity in prevalence between pre- and postoperative depression may be attributed to differences in modalities used to detect depression (different questionnaires vs. psychiatric interviews), differences in parameters used to define depression and differences in post-operative follow-up. Moreover, patients may display somatic symptoms such as fatigue and sleep disturbances which can be difficult to discern from depression (12). Both pre-operative and post-operative depression are underpinned by the same pathophysiological mechanisms and are associated with poor clinical outcomes. Psychiatric assessment should occur pre-operatively and post-operatively. Prior to operating on a patient with pre-operative depression, it may be worthwhile offering additional counseling or support. During the postoperative period, the patient’s psychiatric state should be monitored. Reductions in depression symptoms are expected postoperatively and associated with improvements in quality of life. Conversely, increases in postoperative depression may be attributed to the occurrence of complications or major adverse cardiovascular events (MACE) (13). Hence, there is significant interest in understanding the underlying pathophysiology of depression following cardiac surgery and investigating effective preventative and management modalities within this population.

### 1.1. Risk factors for depression

Risk factors for postoperative depression following cardiac surgery include female gender (5, 14, 15), younger age (16), previous depressive episodes or family history of depression (17) and history of pre-operative depression (18, 19). Social factors such as lower educational levels, lower levels of social support or social isolation also increase the risk of postoperative depression. Social support during the first month following surgery may reduce the likelihood of postoperative depression and impairments in activities of daily living (ADL) at 6 months (20). Naturally, emergency surgery and an extended length of hospital stay contribute to the likelihood of developing postoperative depression (15, 21).

Risk factors for developing preoperative depression are similar to the above, but uniquely include dyspnea upon exertion and at rest (contributing to a higher NYHA classification) (8, 22) and previous myocardial infarction (22). The identification of these contributory factors may allow early recognition of vulnerable patients and early referral for psychiatric assessment.

### 1.2. Screening questionnaires for depression in cardiac surgery patients

The following questionnaires are commonly utilized in depression studies involving cardiac surgery patients.

#### 1.2.1. Patient Health Questionnaire 9

The Patient Health Questionnaire 9 (PHQ-9) is a nine-item self-administered questionnaire used to assess depressive symptoms over the previous 2 weeks using the Diagnostic and Statistical Manual of Mental Disorders 5th Edition (DSM-5) criteria. The PHQ-9 assesses affective, cognitive, and somatic symptoms. Four items are related to somatic symptoms (sleeping difficulties, fatigue, reduced appetite and psychomotor agitation/retardation) (23). The PHQ-9 has high specificity, but low sensitivity for detecting depression in patients with CVD (24, 25). McManus et al. reported a 54% sensitivity and 90% specificity for detecting depression when using a score of ≥10 as the cut-off. This questionnaire may be administered over telephone/telehealth to reliably assess for depressive symptoms (26).

In 2008, the American Heart Association recommends screening for pre-operative depression in patients with coronary heart disease (CHD) with the PHQ-2 or PHQ-9 (preferred). Patients with a positive PHQ-2 screen are asked to complete a PHQ-9 questionnaire, whereas those with a positive PHQ-9 questionnaire should be referred for psychiatric evaluation (27). Patients with a positive PHQ-9 depression screen were at increased risk of MACE at 6 months (OR: 2.16, 95% CI: 0.98–4.74) and five times more likely to receive anti-depressant medication (28). Stenman et al. demonstrated screening with the PHQ-9 in cardiac surgery patients was practically feasible and economically viable, with 64% of elective patients completing the questionnaire prior to surgery (29). Similarly, Gorini et al. reported over an 80% completion rate of the PHQ-9 for pre-operative screening (30). To improve the response rate, the PHQ-9 may be included in the preadmission screen.

#### 1.2.2. Centre for Epidemiological Study of Depression scale

The Centre for Epidemiological Study of Depression (CES-D) is a 20-item self-administered questionnaire which measures common depressive symptoms through a 4-point Likert scale. It comprises an amalgamation of previously validated depression questionnaires. The CES-D has a high internal consistency, as evidenced by a Cronbach’s alpha score of 0.85 (31). For each question, a score of 0 represents minimal symptoms, and a score of 4 represents depressive symptoms most of the time (32). A score greater than 16 indicates clinically significant depression. Uniquely, the CES-D includes several items which assesses interpersonal problems. However, the DSM-5 does not include interpersonal problems within their assessment of depression and interpersonal problems are more likely to occur in psychopathologies such as social anxiety (33).

#### 1.2.3. Beck Depression Inventory

The Beck Depression Inventory (BDI) is a 21-item questionnaire which assesses depressive symptoms over the past 2 weeks. Each answer is recorded on a scale of 0–3, with higher scores indicating more severe depressive symptoms. Studies commonly define depressive symptoms by a score of greater than 10 or greater than 14 (34, 35). A cognitive-affective subscale may be created by adding up the scores from the first 13 items. Conversely, a somatic subscale may be produced by summing the scores from the remaining 8 items (36). Used in over 2,000 studies, the BDI has high internal consistency evidenced by a Cronbach α of 0.82 in a non-psychiatric population (37). The BDI is available and validated in numerous other languages. The BDI is copyrighted, and payment is required to access the forms, which may lead to accessibility issues in resource poor nations (35).

#### 1.2.4. Hospital Anxiety and Depression Scale

The Hospital Anxiety and Depression Scale (HADS) is a 14-question survey, with 7 questions pertaining to depression and anxiety, respectively. Each question is scored between 0 and 3. For the depression subscale, a score less than 8 generally indicates no depression, and a score greater than 11 associated with likely depression (38). The HADS predominantly focuses on cognitive and psychological symptoms rather than somatic features to reduce the potential compounder of somatic features during a hospital admission (39). The HADS was initially designed for the inpatient setting, but can reliably detect anxiety and depression in the primary care and general population (40).

#### 1.2.5. Cardiac Depression Scale

Hare and Davis developed the Cardiac Depression Scale (CDS) specifically for cardiac patients in Australia to account for the range of depressive symptoms, including adjustment disorder with depressed mood. The CDS is a 26-item questionnaire with a Likert scale rating system ranging from 1 to 7, with a score of 1 representing “strongly disagree” while a score of 7 represents “strongly agree.” Higher total scores represent more severe depressive symptoms (41). A CDS cut-off score equal to or greater than 95 provided 97% sensitivity and 85% specificity (42). Findings from the CDS have correlated strongly with the BDI (*r* = 0.73) (41, 43).

#### 1.2.6. Limitations

Self-administered depression screening questionnaires should not replace structured clinical interviews. Several questionnaires, such as the BDI, are restricted by copyright, which may limit access to certain institutions. Additionally, the depression questionnaires should be validated in various languages for different cultures to determine appropriate cut-offs for a positive result. Furthermore, stigma surrounding depression in different cultures may subconsciously influence an individual’s responses to the questionnaires. Finally, studies use different parameter points to define the presence and severity of depression. This makes it difficult to conduct meta-analyses to pool the data for cardiac surgery patients.

### 1.3. Clinical implications of depression

Both pre-operative and post-operative depression with poor clinical outcomes. Psychiatric assessment should occur pre-operatively and post-operatively.

Depression is an independent risk factor for the development of cardiac disease. The risk of death due to cardiovascular disease (CVD) is two times greater in depressed patients (44). The depressed state is associated with poor nutritional choices, limited exercise, tobacco use, and reduce compliance to medications (45–48). This may exacerbate cardiac disease, resulting in the patient requiring surgical intervention. Prior to operating on a patient with pre-operative depression, it may be worthwhile offering additional counseling or support. Preoperative depression is associated with poor functional status (49) poorer quality of life (50), a longer length of hospital stay (*p* < 0.001) (10) and increased level of postoperative pain (51). These patients are less likely to return to work in both a fulltime (OR: 9.43, CI: 3.15–28.21) or part time capacity (OR: 5.44, 95% CI: 1.60–18.53) (52) and are more likely to be re-hospitalized for a cardiovascular cause at 6 months postoperatively (X^2^ = 4.24, *p* < 0.04) (53). Pre-operative depression is also associated with increased mortality following CABG and valve surgery (54–57).

During the postoperative period, the psychiatric state should be monitored. Improvements in physical functioning and quality of life are expected postoperatively and would likely be associated with improvements in depression symptoms. Postoperative depression is associated with higher pain levels up to weeks post discharge (34), lack of functional improvement in patients 6 months post-surgery (58, 59), two fold increased risk of re-admission (60) and substantial risk of atherosclerotic progression (OR 1.50, 95% CI 1.08 to 2.10, *p* = 0.02) (61). Postoperative depression is also associated with increased mortality following coronary artery bypass grafting (CABG) (62). Overall, postoperative depression is an increased 10-year mortality rate following cardiac surgery (HR: 1.8, *p* = 0.04) (44).

## 2. Methods

A literature search using OVID Medline and Embase was performed for studies included in this narrative review. Keywords for depression include depression; pre-operative depression, post-operative depression, depress*. The following keywords for cardiac surgery were included: cardiac surgery; heart surgery; cardiac operation; cardiothoracic surgery; coronary artery bypass graft; CABG; revascularisation surgery; valve replacement and valve repair. Limits included English language, human studies and adults only.

**Figure 1:**
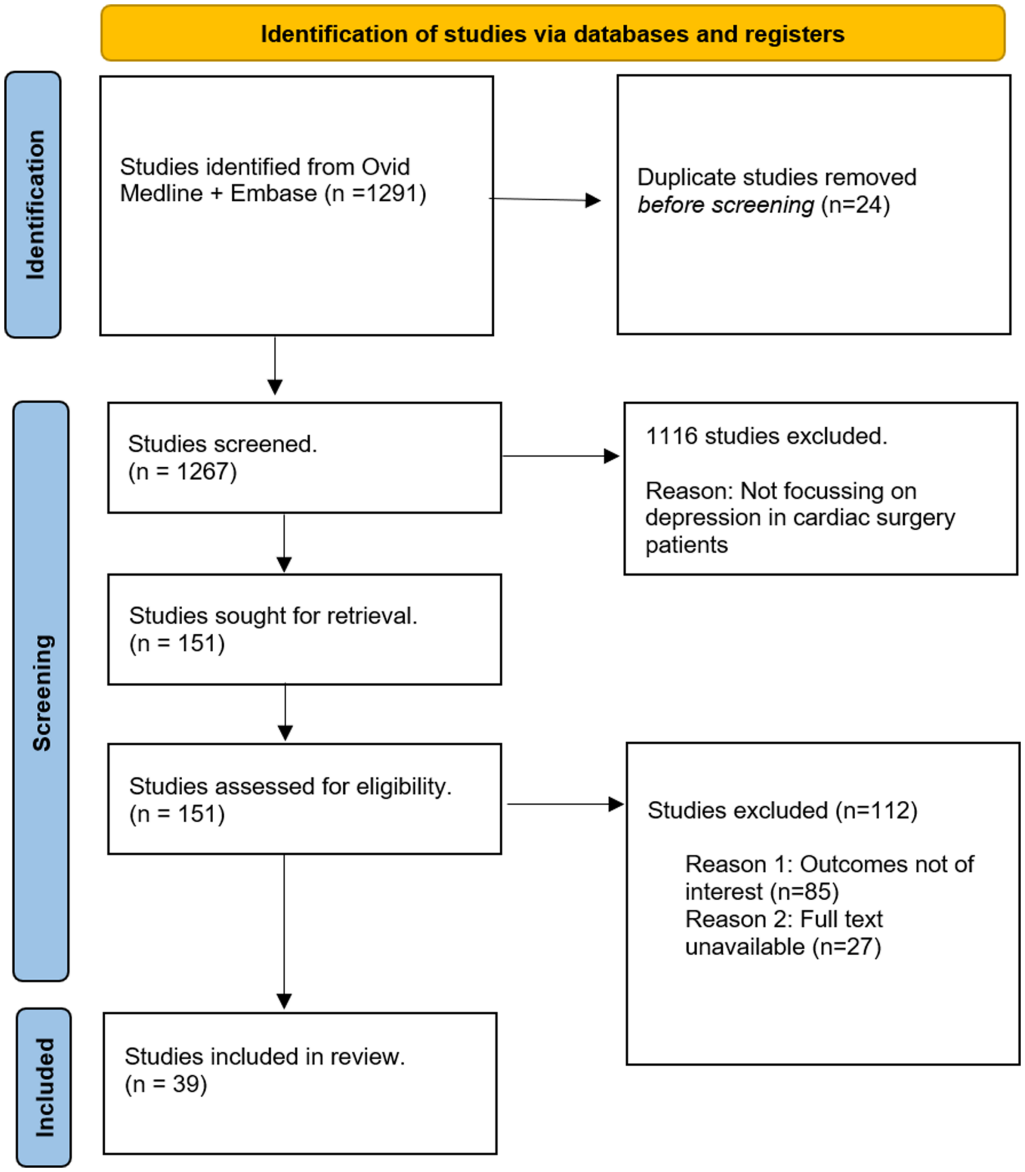
Inclusion of studies for qualitative analysis.

The identification and selection of studies is depicted in Figure 1. Study designs of interest included observational cohort studies and randomized controlled trials. Thirty-nine studies were qualitatively assessed (heart rate variability: 5, inflammation: 4, hypothalamic adrenal axis dysregulation: 2, education: 5, cardiac rehabilitation: 3, cognitive behavioral therapy: 4, prayer: 3, biobehavioral feedback: 1, antidepressants: 7, statins: 2, alternative care: 3). Background information was obtained through references from the search-strategy, and from references within studies.

## 3. Pathophysiology

**Figure 2:**
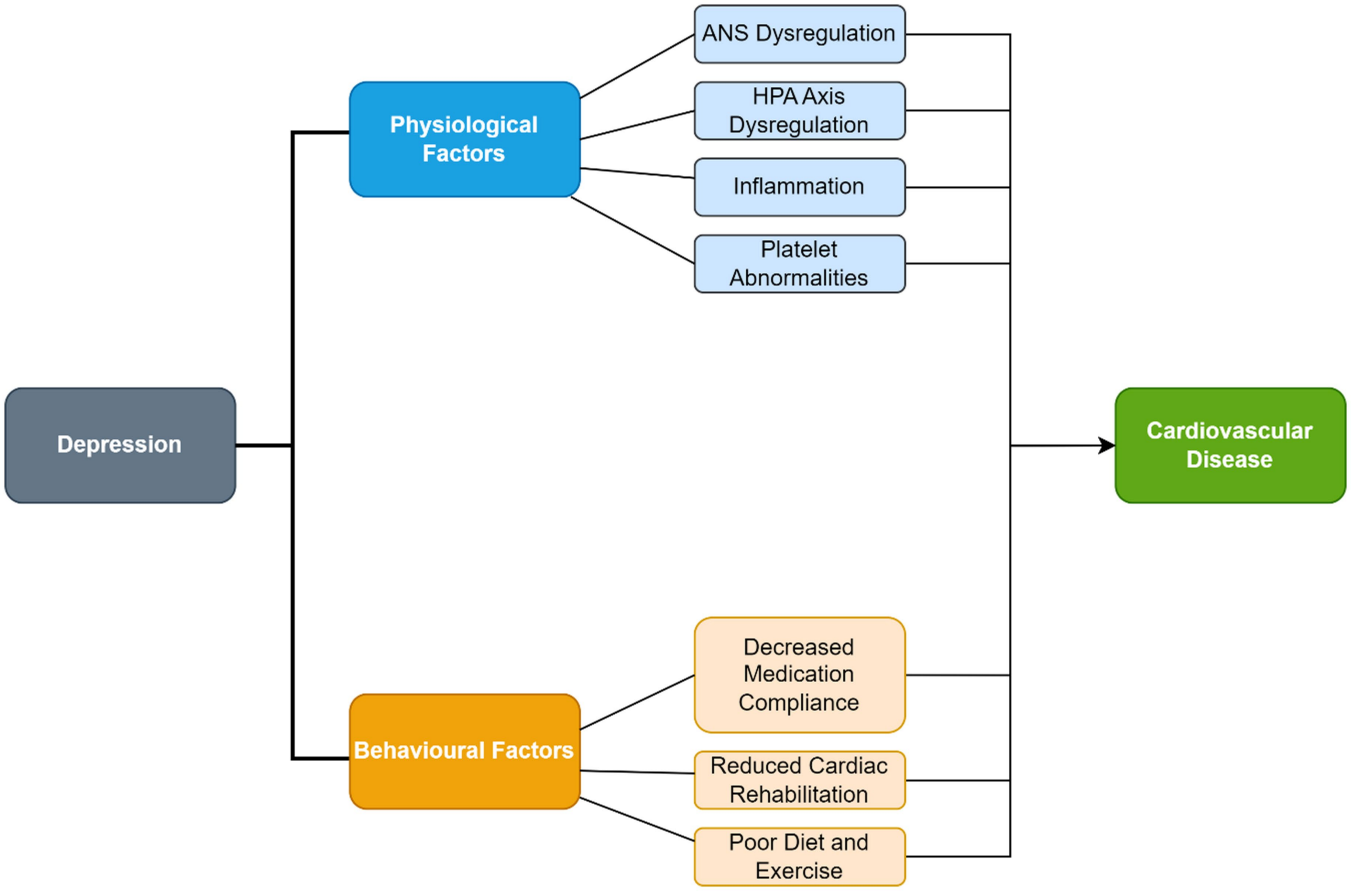
Summary diagram of relationship between depression and coronary heart disease.

The pathophysiology underlying depression in CHD patients includes autonomic nervous system (ANS) dysregulation, inflammation and hypothalamic pituitary adrenal (HPA) axis dysregulation. Surgical trauma may also contribute to postoperative depression through the pathophysiological mechanisms mentioned above. The depressed state may contribute to behavioral factors which may exacerbate CHD. These behavioral and pathophysiological factors are summarized in Figure 2.

### 3.1. Dysregulation of the autonomic nervous system

#### 3.1.1. Overview of heart rate variability

Heart rate may be controlled through sympathetic and parasympathetic mechanisms. Sympathetic autonomic activation increases the heart rate, while parasympathetic activation reduces the heart rate (63). Heart rate variability (HRV) refers to the oscillation in time intervals between heart beats (64). HRV is predominantly recorded using electrocardiography (ECG) and physiological monitors such as the Nexus 10 BioTrace equipment. Generally, during the recording process, patients are asked to keep their eyes open and keep their wrists still. Occasionally, the investigator will read pleasant travel excerpts to keep patients relaxed. This standardized process has been previously shown to mimic normal waking states of arousal (65). Moreover, patients are usually excluded from studies if they are not in sinus rhythm, given the increased difficulty in determining HRV (66).

Physiologically, HRV reflects the overall balance between the sympathetic and parasympathetic systems on the cardiovascular system (67, 68). A higher HRV is generally indicative of a well-functioning autonomic nervous system that is responsive to physiological and psychological stressors. Conversely, a low HRV may represent excessive sympathetic nervous system activation, or inadequate vagal tone (64). Additionally, a low HRV may predict acute cardiac complications and sudden cardiac death in patients with acute myocardial infarction and major pulmonary resections (69, 70).

HRV may be calculated using the time domain, or the frequency domain (64). The time domain measurements of HRV quantify the time between heart beats. HRV may be calculated as the standard deviation of the mean R-R or N-N interval (SDNN) on ECG in milliseconds. The SDNN accounts for cyclical components responsible for HRV such as respiration and blood pressure fluctuations (71). Additionally, HRV may also be calculated as the root of the mean square differences in the N-N intervals (rMSSD). This parameter represents the short-term heart rate variability and acts as an index of vagal outflow (72). Power spectral analysis can quantify the contribution of autonomic cardiac regulation to HRV using the knowledge of frequency and power (the energy signal found within a frequency band) (73). Very Low Frequency (VLF) power is defined as <0.04 Hz, Low Frequency (LF) power is between 0.04–0.15 Hz and High Frequency (HF) power is between 0.15–0.40 Hz HF power represents parasympathetic activity, whereas LF power represents sympathetic and some parasympathetic activity (64). Thus, a ratio of LF / HF (ms^2^/ms^2^) assesses overall autonomic balance.

An increase in the LF/HF ratio suggests sympathetic dominance, whereas a decrease reflects a parasympathetic dominance (74, 75). Autonomic dysregulation may result in electrical instability of myocytes and predispose patients to arrhythmias, myocardial ischemia, and sudden cardiac death (76, 77). Depressed patients exhibit features of ANS dysregulation through elevations in plasma and urinary catecholamines such as noradrenaline, decreased HRV and an elevated basal HR compared to non-depressed patients (78, 79). Consequently, there is a likely link between CHD, depression and ANS dysregulation.

#### 3.1.2. Depression reduces vagal outflow following cardiac surgery

Depression affects the sympathovagal balance following cardiac surgery, specifically with reduced vagal outflow. Studies assessing the pathophysiology of depression in the cardiac surgery population are summarized in Table 1.

**Table 1:** Studies assessing the association between autonomic dysregulation and depression in the cardiac surgery population.

Study	Aim	Surgery type	Study type	Patient number	Patient groups	Intervention	Definition of depression	Findings
Dao et al. (80)	To assess the potential mechanism between autonomic dysregulation and depression in CABG patients	CABG ± valve procedure	Prospective observational study	*n* = 180	CHD + depression No CHD + depression CHD + no depression No CHD + no depression	Measure HR and HRV after 12 h fast, and assessment of plasma noradrenaline	Diagnosis of MDD as per MINI	CHD + depressed patients had a lower heart rate variability compared to patients with CHD and no depression (OR: 0.597, 95% CI: 0.497–0.718) CHD + depressed patients had a higher heart rate compared to patients with CHD and no depression (OR: 1.12, 95% CI: 1.02–1.04)
Patron et al. (81)	To assess the association between depression and heart rate variability following cardiac surgery	Cardiac surgery (not defined)	Prospective observational study	*n* = 33	Depressed (*n* = 11) Non-depressed (*n* = 22)	Assessment of HR and HRV	CES-D > 16	Depressed patients had a reduced HRV, as calculated by SDNN, in patients with depression following cardiac surgery (17.5 ms vs. 36.7 ms, *p* = 0.02).
Patron et al. (82)	To assess the relationship between depression, emotional regulation and autonomic dysfunction following cardiac surgery	Cardiac surgery (not defined)	Prospective observational study	*n* = 68	Depressed (*n* = 25) Non-depressed (*n* = 43)	Assessment of affective evaluation through a questionnaire, followed by HR and HRV recording	CES-D > 16	Depressed patients were more likely to use emotional suppression strategies and this may mediate sympathovagal imbalance [*F*(1, 65) = 5.31, *p* < 0.03]
Patron et al. (83)	To assess whether unpleasant imagery may mediate ANS dysregulation in depressed patients who have undergone cardiac surgery	Cardiac surgery (not defined)	Prospective observational study	*n* = 28	Depressed (*n* = 14) Non-depressed (*n* = 14)	Patients completed an emotional imagery test including listening to a pleasant, neutral and unpleasant script. HR and HRV are measured following	CES-D > 16	Depressed patients had a greater reduction in high frequency power during unpleasant emotional imagery (*p* = 0.003, Cohen’s *d* = 1.34)
Gentili et al. (84)	To assess whether a multi-feature analysis can predict the CES-D score of patients and automatically classify them as depressed or non-depressed.	CABG ± valve procedure	Prospective observational study	*n* = 31	Single group of post cardiac surgery patients	Authors developed a model to predict CES-D scores	CES-D > 16	The multi-variate model predicted the CES-D score in all 31 patients, with a variance of 89.93%. The model could also discriminate between whether patients were depressed or non-depressed with 86.75% accuracy.

CABG, coronary artery bypass graft; CES-D, centre for epidemiological studies; CHD, coronary heart disease; HR, heart rate; HRV, heart rate variability.

Dao et al. proposed autonomic cardiovascular dysregulation as a potential mechanism in the development of depression following cardiac surgery (80). ANS dysregulation was defined as a high basal HR, low HRV and high levels of plasma norepinephrine. The depressed + CABG group had a significantly lower HRV and higher basal HR compared to the other groups, but there were no differences in the level of plasma norepinephrine. This lack of difference may be reflective of the autonomic state of the arm rather than systemically, as blood was taken from the cubital fossa. Additionally, patients with depression + CABG had a longer length of stay compared to non-depressed patients. This suggests that autonomic dysregulation resulting from depression may negatively affect CABG outcomes.

Within the cardiac surgery population, ANS dysregulation is driven by an attenuated vagal response. In a study of 33 patients, Patron et al. demonstrated a reduction in HRV as calculated by SDNN in patients with depression (CES-D greater than16) following cardiac surgery (17.5 ms vs. 36.7 ms, *p* = 0.02) (81). Depressed cardiac surgery patients exhibited an attenuated vagal response, as demonstrated by a reduced high-frequency power and subsequently an increased LF/HF ratio (Depressed: 3.6 vs. non-Depressed: 0.9, *p* = 0.08). This suggests reduced vagal outflow, rather than sympathetic hyperactivity, is the corresponding factor between depression and CHD in the cardiac surgery population. Anxiety may also be associated with a reduction in HRV (85). However, Patron et al. demonstrated HRV reductions in depressed patients following cardiac surgery was independent of anxiety, using the STAI Y1 and STAI Y2 tests.

The mechanisms underlying a reduction in HRV in depressed patients following cardiac surgery is poorly understood. Patron et al. assessed whether emotional regulation strategies such as cognitive reappraisal or emotional suppression contributed to autonomic dysfunction in depressed patients following cardiac surgery. Cognitive reappraisal refers to the reframing of an emotion-provoking situation to a neutral thought (86). Conversely, emotional suppression strategies refer to the self-inhibition of negative and intrusive thoughts (87). Within this study, depressed patients were more likely to use emotional suppression strategies, and this coping mechanism was associated with autonomic dysfunction following cardiac surgery (82). Additionally, Patron et al. examined the effect of pleasant, neutral or unpleasant emotional imagery on depressed patients following cardiac surgery. They found depressive patients had increased vagal withdrawal in response to unpleasant emotional imagery, as measured by high-frequency power (*p* = 0.003) (83). These results complement the literature, and suggest depressive patients demonstrate mood congruent bias and are more likely to react negatively to negative stimuli (88, 89).

Gentili et al. developed a regression model using HRV features in time and frequency, as extracted from five-minutely ECG recordings to predict cardiac surgery patients’ CES-D score, and consequently the presence and severity of depressive symptoms. The model could predict the CES-D score in all 31 patients, with a variance of 89.93%. Additionally, they could discriminate whether patients were depressed or non-depressed with 86.75% accuracy (84). This model should be tested, and validated in a larger population. If successful, it may be feasible to collect HRV measurements automatically at the bedside and use this parameter as a screening tool to detect patients with depression. This would be a useful tool in hospitals where psychological evaluation of patients following cardiac surgery is unavailable.

### 3.2. Inflammation

#### 3.2.1. Link between inflammation and depression in cardiac surgery patients

There is a bidirectional relationship between inflammation and depression (90). In the cardiac surgery population, inflammation drives the pathophysiology of the underlying CHD but may also manifest because of cardiac surgery itself. This pro-inflammatory state may affect serotonergic neurotransmission through the Kynurenine pathway and contribute to depression (91). Pro-inflammatory cytokines within the peripheries may enter the brain by crossing the blood–brain barrier, or indirectly facilitate activation of microglia within the brain. Hence, excessive peripheral inflammation may contribute to neuroinflammation and potentially to depression (92, 93). On the contrary, depression may contribute to inflammation through the upregulation of pro-inflammatory cytokines and acceleration of the atherosclerotic process (94).

##### 3.2.1.1. Inflammatory nature of coronary heart disease

Atherosclerosis is a chronic inflammatory process which leads to the buildup of plaque within the arterial wall (95). Atherosclerosis is the main pathophysiological contributor to CHD. The pathogenesis of atherosclerosis involves the following key steps: endothelial damage and the formation of foam cells, fatty streaks, intermediate lesions, atheroma and finally, atherosclerotic plaques (96, 97).

Firstly, endothelial insults may be caused by reactive oxygen species, turbulent blood flow (particularly at arterial branch points), hyperglycemia and hyperlipidemia. Low-density lipoproteins may penetrate the injury site and migrate into the tunica intima (98). This triggers a pro-inflammatory process which upregulates the expression of cell-adhesion molecules such as VCAM-1 upon the endothelial surface (99). Monocytes adhere to the endothelial surface and migrate into the subendothelial spaces. Subsequently, the macrophages phagocytose oxidized low-density lipoprotein to form foam cells. The accumulation of foam cells leads to fatty streaks, which may remain stable and ultimately form atherosclerotic plaques or regress (100).

##### 3.2.1.2. Association between inflammation and cardiac surgery

During cardiac surgery, factors which may contribute to a systemic inflammatory response include surgical trauma, blood surface interactions with the cardiopulmonary bypass circuitry, endotoxemia, and ischemic reperfusion injuries. These initiating factors trigger a series of responses from the complement system, neutrophils, cytokines, the coagulation cascade, and the vascular endothelium which result in a systemic inflammatory response. In addition, excessive inflammation may cause organ dysfunction (101–103).

##### 3.2.1.3. The kynurenine hypothesis of depression

Tryptophan is an essential amino acid with two predominant fates – conversion into kynurenine or 5-hydroxytryptamine (serotonin) (104, 105). Over 95% of consumed dietary tryptophan undergoes degradation through the kynurenine pathway (106). The conversion of tryptophan into kynurenine is catalyzed by hepatic tryptophan 2,3-dioxygenase (TDO) and extra-hepatic Indolamine 2,3-dioxygenase (IDO) (107).

From here, kynurenine has three possible fates. Firstly, within microglia, kynurenine may be consumed to facilitate the formation of 3-hydoxykynurenine and its metabolites 3-hydroxyanthranilic acid and quinolinic acid. Quinolinic acid is a glutamate N-methyl-D-aspartate (NMDA) agonist which has excitotoxic and neurotoxic properties associated with depression (108–110). Secondly, also within microglia, kynurenine may also be converted to anthranilic acid, which may be implicated in depression or schizophrenia (111–113). Thirdly, kynurenine may be consumed within the skeletal muscles or peripheries to form kynurenic acid. Kynurenic acid has neuroprotective, antidepressant and anticonvulsant properties, by acting as a non-competitive NMDA receptor blocker (114, 115).

In stressful and pro-inflammatory states, tryptophan is preferentially converted to kynurenine. This is represented by an elevated plasma kynurenine to tryptophan ratio (K/T ratio) (116). An elevated K/T ratio has been observed in CHD depressive patients (117). Perturbations in the metabolism of tryptophan have also been associated with metabolic syndrome risk factors, such as hypertension, obesity and dyslipidemia (91). Inflammation suppresses intrahepatic TDO, while extrahepatic IDO is expressed. Hence, inflammation is associated with an increased production of neurotoxic substrates from the kynurenine pathway which may contribute to depression (118). Moreover, the shunting of tryptophan down the kynurenine pathway depletes serotonin levels, supporting the monoamine hypothesis of depression whereby low serotonin levels contribute to low mood (119). Consequently, there is significant interest in examining inflammation as a pathophysiological mechanism in driving depression in cardiac surgery patients.

#### 3.2.2. Commonly assessed inflammatory markers

Psychological and psychosocial stressors facilitate the release of pro-inflammatory cytokines (120). Magnocellular neurons are sensitive to stress and neuroendocrine changes and facilitate the release of pro-inflammatory cytokines into the general circulation via the neurohypophysis (121). Studies have primarily assessed whether there is a relationship between the peripheral concentration of pro-inflammatory markers such as c-reactive protein (CRP), interleukin-6 (IL-6) and tumor necrosis factor-α (TNF-α) and the development of depression. Elevated inflammatory markers may also attenuate the response to antidepressant therapy (122, 123).

CRP is an acute phase reactant which increases during inflammation (124, 125). CRP is an established cardiovascular risk factor, with higher levels associated with poorer cardiovascular outcomes (126). A large proportion of studies assessing the association between inflammation and depression use CRP as their marker of inflammation. IL-6 is a multifunctional cytokine involved in immunological processes such as hematopoiesis, immune modulation and acute inflammation (127, 128). It is produced by numerous cells including macrophages, neutrophils, and lymphocytes (129). Given its multisystem involvement, IL-6 has been previously suggested to play a large role in the pathophysiology of depression (130). Excessive levels of IL-6 may affect the HPA axis and may be associated with left ventricular dysfunction and poor outcomes following open cardiac surgery (131, 132). Studies of inflammation and depression in the cardiac surgery population are summarized in Table 2.

**Table 2:** Studies assessing the association between inflammation and depression.

Study	Aim	Surgery type	Study type	Patient number	Patient groups	Intervention	Definition of depression	Findings
Yang et al. (133)	To assess the contribution of preoperative high sensitivity CRP levels on the development of both pre and post-operative depression	Elective CABG	Prospective observational study	*n* = 232	No pre-operative depression (*n* = 190) Pre-operative depression (*n* = 42)	PHQ completed 3 days before surgery and 6 months postoperatively. High sensitivity CRP collected at baseline	PHQ ≥ 10	Each standard deviation increase in preoperative logarithmically transformed high sensitivity CRP levels was associated with an increased odds of developing pre-operative depression (OR: 1.16, *p* = 0.001) and postoperative depression at 6 months (OR: 1.15, *p* = 0.002).
Poole et al. (134)	To assess the association between postoperative CRP, depressive features and length of hospital stay	CABG ± valve procedure	Prospective observational trial	*n* = 145	No pre-operative depression (*n* = 100) Pre-operative depression (*n* = 45)	Postoperative CRP measured between day1-3, then between day 4 and 8. BDI assessed prior to surgery	BDI ≥ 10	Patients with pre-operative depression are 3.5x more likely to stay in a hospital for a week following surgery (*p* = 0.007) Every unit of increase in high sensitivity CRP measured between postoperative day 1–3 increased the odds of an increased length of stay by 1% (*p* = 0.030) Elevations in the level of CRP measured between postoperative days 4–8 significantly contributed to depression status and longer hospital stay (*t* = 2.62, *p* = 0.010)
Ivankovic et al. (135)	To assess the contribution of both pre and post-operative CRP levels to preoperative depression symptoms and length of hospital stay	Elective CABG	Prospective observational trial	*n* = 212	No pre-operative depression (*n* = 186) Pre-operative depression (*n* = 26)	Postoperative CRP measured between day1 and 3, then between day 4 and 6. BDI assessed prior to surgery	BDI ≥ 13	73.1% of patients with pre-operative depression had a length of stay over 7 days. In comparison, 56.5% of patients without preoperative depression had a length of stay over 7 days. Following stratification of preoperative BDI scores into depressed or non-depressed (BDI < 10), there was no association between binary BDI scores and length of postoperative hospital stay. Post-operative CRP measured at postoperative day 4–6 was associated with a prolonged hospital stay (OR: 1.017, 95% CI: 1.005–1.029, *p* = 0.009). No association detected between pre-operative BDI scores and CRP levels
Streptoe et al. (136)	To assess the association between the level of inflammatory markers and depressive symptoms	Elective CABG	Prospective observational trial	*n* = 145	Not specified	Depressive symptoms measured 1 month prior to surgery and 12 months following surgery Plasma IL-6 and Interferon gamma were measured between postoperative days 1–3	BDI cut off not specified	Early postoperative IFN-γ levels which were in the upper tertile (mean concentration: 56.68 ± 7.5 pg./mL) was associated with depressive symptoms at 12 months (OR: 4.32, *p* = 0.024). In contrast, there was no association between IL-6 and the development of postoperative depressive symptoms

BDI, beck depression inventory; CABG, coronary artery bypass graft; CRP, c reactive protein; IFN- γ; interferon gamma; IL-6, interleukin-6; PHQ, patient health questionnaire.

#### 3.2.3. Inflammatory markers may predict depression following cardiac surgery

Studies assess different inflammatory markers on the development of depression, making it difficult to draw definitive conclusions about their effect. Measuring the levels of CRP may identify patients at risk of developing depression following cardiac surgery. Yang et al. reported that for an increase in each standard deviation of logarithmically transformed high sensitivity pre-operative CRP, there was an increased odds of developing depression pre-operatively (OR: 1.16, *p* = 0.001) and at 6 months post-operatively (OR: 1.15, *p* = 0.002). This relationship was maintained even after adjusting for confounding variables and risk factors such as gender, education level, medications such as statins, and occurrence of major cardiac adverse events (133). Similarly, Poole et al. demonstrated that elevations in the level of CRP measured between postoperative days 4–8 significantly contributed to the depression status and longer hospital stay (t = 2.62, *p* = 0.010) (134). In contrast, Ivankovic et al. did not demonstrate a relationship between preoperative depression status and postoperative CRP levels (135).

Additionally, Streptoe et al. reported that early postoperative interferon γ (IFN-γ) levels which were in the upper tertile (mean concentration: 56.68 ± 7.5 pg./mL) was associated with depressive symptoms at 12 months (OR: 4.32, *p* = 0.024) (136). In contrast, there was no association between IL-6 and the development of postoperative depressive symptoms. Previous studies have demonstrated a correlation between the level of IL-6 and development of depressive symptoms (130, 137). IFN-γ is directly involved in the induction of indoleamine 2,3-dioxygenase, which is involved in the catabolism of tryptophan to products such as kynurenine (138). Elevated concentrations of kynurenine increase the likelihood of deleterious downstream effects as mentioned previously.

#### 3.2.4. Relationship between depression, inflammatory markers, and length of hospital stay

##### 3.2.4.1. Association between depression and length of hospital stay

The association between depression status and length of hospital stay following cardiac surgery is unclear. Length of hospital stay is a common proxy measure of acute physical recovery (139). Poole et al. reported that patients with elevated preoperative depressive symptoms (BDI greater than 10) prior to CABG were significantly more likely to stay in the hospital for longer than a week, compared to non-depressed patients (OR 3.51, 95% CI: 1.415–8.693, *p* = 0.007) (134). This relationship is likely mediated by an elevation in the level of CRP measured between postoperative days 4–8 (*t* = 2.62, *p* = 0.010). These results suggest pre-operative depression may promote excessive inflammation and lead to poorer outcomes and extended hospital stays. On the contrary, Ivankovic et al. did not detect a significant association between elevated preoperative depression scores (BDI greater than 13) and extended postoperative length of stay (greater than 7 days) (135). Additionally, within the same study, Ivankovic and colleagues subsequently stratified the patients’ preoperative BDI scores into depressed or non-depressed by applying the same cutoff as Poole et al. (Non-depressed: BDI less than 10; depressed: BDI greater than 10). Even with this filter, Ivankovic did not detect a significant association between binary BDI scores, and the length of postoperative hospital stay. The average EuroSCORE in Poole et al., study was 4.21 ± 2.79, while the median in Ivankovic et al.’s study was 1.1 with a range between 0.7 and 2.0. This may have contributed to the differences in postoperative recovery and length of hospital stay.

### 3.3. Disruption of the hypothalamic pituitary adrenal axis

#### 3.3.1. Overview of the HPA axis

Consisting of the hypothalamus, pituitary gland and adrenal gland, the HPA axis is responsible for regulating the mammalian stress response, immunity, metabolic functioning, neurogenesis, neuronal survival and the emotional appraisal of events (140). Stressful stimuli trigger the production of corticotrophin-releasing hormone (CRH) and arginine vasopressin (AVP) by the paraventricular nucleus of the hypothalamus (141). CRH is a 41 amino acid peptide found within the central nervous system (142). Conversely, AVP is a cyclic nonapeptide with two forms, with one responsible for blood pressure regulation and the other responsible for the stress response (143). Following the binding of CRH to the CRH receptors within the anterior pituitary, adrenocorticotrophic hormone (ACTH) is released. Subsequently, ACTH will travel via the blood stream to act on the adrenal gland receptors to facilitate the release of glucocorticoids such as cortisol, mineralocorticoids such as aldosterone and androgens such as testosterone (144).

The HPA axis is regulated through a negative feedback mechanism. High concentrations of cortisol inhibit the further release of CRH and ACTH from the hypothalamus and pituitary gland, respectively, (145). Rapid increases in the concentration of cortisol also inhibit the HPA axis (146). On the contrary, glucocorticoids have been demonstrated to increase the concentration of CRH in limbic regions such as amygdala (147). Elevated expression of CRH within the limbic region has been associated with depressive symptoms (148, 149).

#### 3.3.2. Dysregulation of the HPA axis in depression

Dysregulation of the HPA axis is commonly seen in MDD. Firstly, chronic emotional stress and MDD is associated with elevated cortisol secretion (150, 151). Normally, the HPA axis reacts appropriately through negative feedback mechanisms and reduce excess cortisol secretion. However, with HPA axis dysregulation, the negative feedback loop is impaired by reduced sensitivity of glucocorticoid receptors. This leads to hyperactivity of the HPA axis and further secretion of cortisol (152, 153). Cortisol hypersecretion is associated with depression following cardiac surgery (154). Poole et al. demonstrated a steeper change in cortisol concentration throughout the day (steeper cortisol slope) measured at 2 months post CABG is associated with a reduced odds of developing depression (defined as BDI greater than 10) at 12 months following surgery (OR: 0.661, 95% CI: 0.437–0.998, *p* = 0.049) (155).

Elevated CRH concentrations are observed in MDD, but not in psychopathologies such as schizophrenia and bipolar disorder (156, 157). Upon resolution of MDD, the CRH levels appear to normalize (158). Additionally, in response to exogenous CRH administration, there is a reduction in ACTH secretion (159–161). This may be attributed to the downregulation of CRH receptors within the hypothalamus. Disturbances in the HPA axis may lead to structural changes to the effectors of this axis. Hypersecretion of CRH due to impairment of the negative feedback loop may lead to pituitary hypertrophy (162, 163). Hippocampal changes in response to MDD have been an area of interest given this structure contains a high concentration of glucocorticoid receptors. A reduction in hippocampal volume is associated with the chronicity of disease, the severity and frequency of MDD episodes (164, 165). Reports of alterations to the size of the amygdala in MDD has been conflicting (166–168). HPA axis hyperactivity is associated with the development of classic cardiovascular risk factors including hypertension, dyslipidemia, impaired glucose tolerance and truncal obesity (169, 170). Hence, HPA axis dysregulation is a likely link between depression and cardiovascular disease.

#### 3.3.3. Effect of dexamethasone on depression following cardiac surgery

Dexamethasone is a glucocorticoid receptor agonist with anti-inflammatory properties. Prolonged use is associated with neuropsychiatric deficits such as postoperative cognitive dysfunction, depressive symptoms and mania (171). Studies of dexamethasone in the cardiac surgery population are summarized in Table 3. Kok et al. assessed the effectiveness of administering a single intraoperative dose of intravenous dexamethasone, a glucocorticoid receptor agonist, on depression and post-traumatic stress disorder (PTSD) following cardiac surgery (172). Compared to the placebo, a single intraoperative dose of dexamethasone (1 mg/kg of bodyweight up to a maximum of 100 mg) did not affect the overall prevalence of depression following cardiac surgery. The overall rates of PTSD were similar between the groups. However, dexamethasone demonstrated long-lasting protective effects against the development of depression and PTSD in women. Six women who received dexamethasone developed depression, while 20 women who received placebo developed depression (*p* < 0.003). Similarly, 4 women who received dexamethasone developed PTSD, while 16 women who received placebo developed PTSD (*p* < 0.004). Women are more likely to be affected by HPA axis dysregulation and to have a higher basal cortisol concentration (174, 175). Additionally, gender may differentially affect HPA axis activation and glucocorticoid sensitivity, which in turn modulates the pro-inflammatory cytokine production (176, 177).

**Table 3:** The effect of dexamethasone on improving depression in cardiac surgery patients.

Study	Aim	Surgery type	Study type	Patient number	Patient groups	Intervention	Definition of depression	Findings
Kok et al. (172)	To assess the effectiveness of administering a single intraoperative dose of dexamethasone against preventing depression and PTSD following cardiac surgery	Assorted cardiac surgery (CABG or valves)	Follow up of randomized controlled trial	*n* = 1,125	Dexamethasone (*n* = 561) Placebo (*n* = 564)	Patients received a high intraoperative dose of dexamethasone (1 mg/kg) or placebo	BDI >13.5	69 patients receiving dexamethasone developed depression, whereas 78 patients receiving placebo developed depression (OR: 0.92, 95% CI: 0.64–1.31). Six women who received dexamethasone developed depression, while 20 women who received placebo developed depression (*p* < 0.003). Similarly, 4 women who received dexamethasone developed PTSD, while 16 women who received placebo developed PTSD (*p* < 0.004).
Kok et al. (173)	To assess whether common hypothalamic pituitary adrenal axis polymorphisms would protect against the development of PTSD and depression following dexamethasone	Assorted cardiac surgery (CABG or valves)	Follow up of randomized controlled trial	*n* = 996	Single group who completed were enrolled in the dexamethasone for cardiac surgery randomized controlled trial	Patients received a high intraoperative dose of dexamethasone (1 mg/kg) or placebo Genotyping assessed rs41423247, rs10052957, rs6189, rs6195 and rs6198.	Not specified	Did not identify polymorphisms which conferred protection against depression following cardiac surgery. On the contrary, three single nucleotide polymorphisms in the glucocorticoid receptor were required for dexamethasone to exert its protective effects against PTSD, including the rs41423247, rs10052957 and the rs6189 polymorphisms

BDI, beck depression inventory; CABG, coronary artery bypass graft; PTSD, post-traumatic stress disorder.

Genetic polymorphisms in the glucocorticoid receptor may significantly influence an individual’s susceptibility to develop depression and dictate their therapeutic response to antidepressant therapy (178, 179). Kok et al. assessed five common, single nucleotide polymorphisms on the glucocorticoid receptor including: rs41423247, rs10052957, rs6189, rs6195, and rs6198. They not identify polymorphisms which conferred protection against depression following cardiac surgery. On the contrary, three single nucleotide polymorphisms in the glucocorticoid receptor were required for dexamethasone to exert its protective effects against PTSD, including the rs41423247, rs10052957, and the rs6189 polymorphisms (173). The glucocorticoid receptor single nucleotide polymorphism rs6189 has been associated with reduced glucocorticoid receptor sensitivity and a faster response to treatment in MDD patients (180, 181). Thus, it is unclear why the protective effects of dexamethasone did not interact with this glucocorticoid receptor in the study. This is the largest study to date examining the genetic variability of the HPA axis in response to the administration of intraoperative dexamethasone in the cardiac surgery population. Future studies should also assess the concentration of CRH, ACTH and serum/salivary cortisol to gain a holistic view of HPA axis functioning.

## 4. Preventative and management strategies

**Figure 3:**
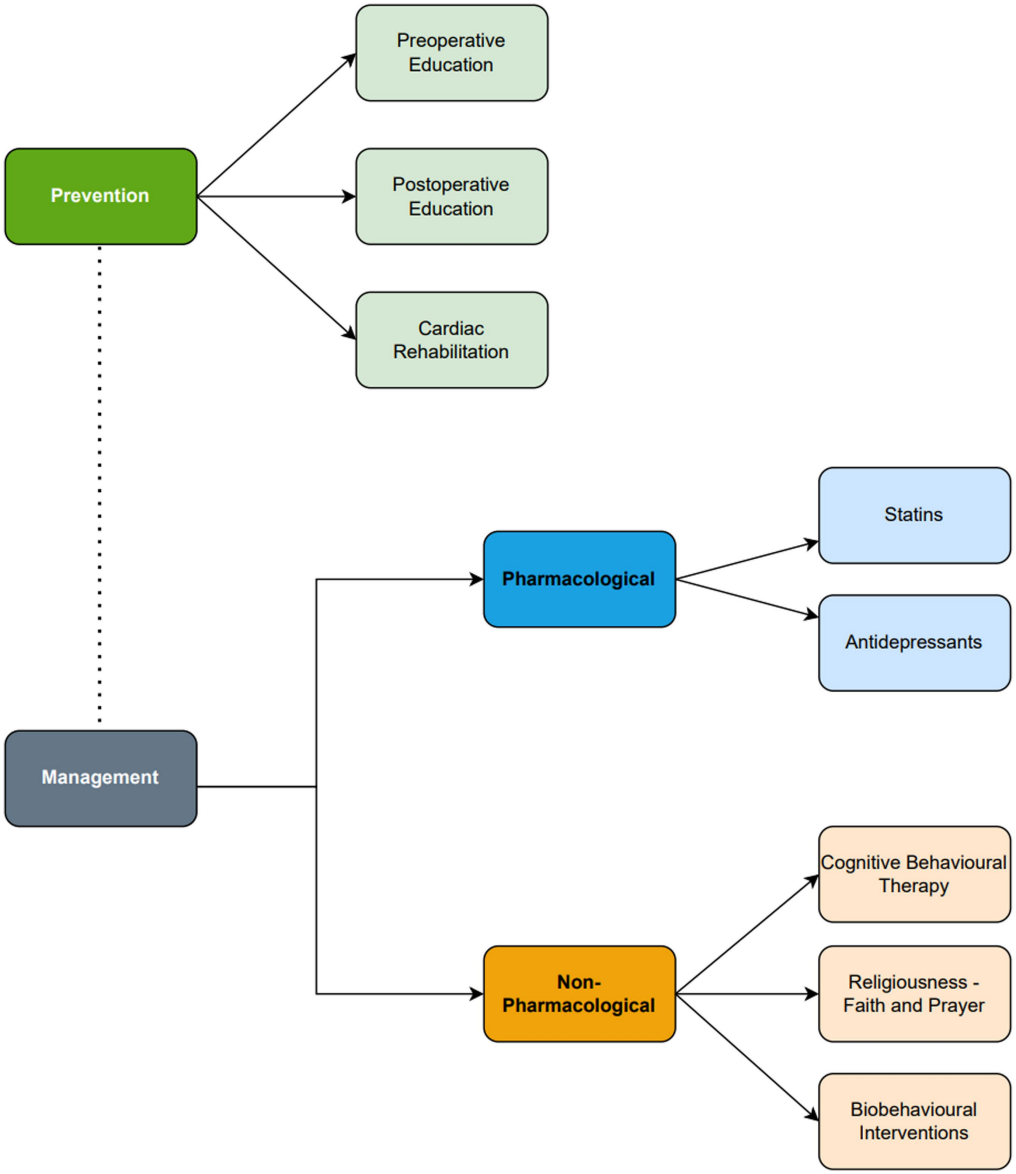
Summary diagram of the prevention and management of depression following cardiac surgery. Antidepressant and cognitive behavioral therapy are the core management for depression following cardiac surgery. Statins, religiousness/spirituality and biobehavioral interventions are emerging adjuncts which should only be considered in clinical practice following further evidence.

The preventative and management strategies for depression following cardiac surgery are summarized in Figure 3. Management strategies may be divided into pharmacological or non-pharmacological strategies.

### 4.1. Preventative strategies

#### 4.1.1. Preoperative education

Preoperative education is effective in reducing depressive symptoms following cardiac surgery. Education may be delivered at the bedside, in a group, via phone call, or even through social media platforms. Long wait times for surgery, particularly in the elective setting, contributes to mental distress (182, 183). Preoperatively, patients should be informed of the surgical process and risks involved with the procedure, required investigations, secondary prevention through risk factor modification as well as any other concerns or expectations. Patients should also be informed of whether surgical access will be achieved via median sternotomy or through minimally invasive methods (e.g., lateral thoracotomy). Access via median sternotomy is known to cause anxiety and addressing this concern may reduce the risk of anxiety and depression (184).

Preoperative education should also involve psychological support and setting health and recovery expectations (185). Conversely, postoperative education should focus on the recovery and rehabilitation process. Centers should provide both preoperative and postoperative education to reduce depression and anxiety. The results of educational interventions in the cardiac surgery setting are discussed below.

##### 4.1.1.1. Preoperative education alone

Three studies were identified which examined preoperative education alone (Table 4). Two of the studies supported preoperative education in reducing depressive symptoms. In an RCT of a Chinese population, Guo et al. reported that compared to usual care, preoperative education was associated with a significant reduction in depressive symptoms at 7 days postoperatively as measured by HADS (Mean Difference: 2.1, 95% CI: −3.19 to −0.92, *p* < 0.001) (186). Their preoperative education intervention involved the distribution of a flyer and an accompanying explanation of what to expect from preadmission through to surgery and postoperative recovery. In comparison, the usual care group received general advice from the surgeon and anesthetist the day prior to their surgery. Preoperative education may have benefitted this population for several reasons. Firstly, patients undergoing cardiac surgery in several Chinese hospitals are admitted 1 week pre-operatively, contributing to the build-up of anxiety and depressive symptoms. Secondly, Chinese cardiac patients tend to be less informed about the finer details of their procedure. A qualitative analysis revealed surgeons generally discussed the severity of cardiac disease with the patient, but not the risks of surgery (which they limited to the patients’ family members) (189). However, surgeons should discuss risks with patients, as one reported “knowledge is comforting, with the more [they] knew, the less anxious [they] became” (190). Patients undergoing cardiac surgery should be placed in nearby beds to allow patients to support each other.

**Table 4:** The effect of preoperative education on improving depression following cardiac surgery.

Study	Aim	Surgery type	Study type	Patient number	Patient groups	Intervention	Definition of depression	Findings
Guo et al. (186)	To assess whether preoperative education is effective in reducing anxiety and depression in Chinese cardiac surgery patients	Elective cardiac surgery (CABG, valve or congenital surgery)	Randomized controlled trial	*n* = 135	Usual care (*n* = 67) Preoperative education (*n* = 68)	Patients in the preoperative intervention group received an information booklet about heart surgery and verbal advice at around 2–3 days prior to surgery Usual care group received unstructured verbal advice	Not specified, but measured using HADS	Preoperative education was associated with a significant reduction in depressive symptoms at 7 days postoperatively as measured by HADS (Mean Difference: 2.1, 95% CI: −3.19 to −0.92, *p* < 0.001)
Furze et al. (187)	To assess the effect of a brief, home based cognitive behavioral program (HeartOp) compared to only preoperative nurse counseling	Elective first time CABG	Randomized controlled trial	*n* = 204	HeartOp group (*n* = 100) Control group (*n* = 104)	HeartOp programme discusses cardiac myths and misconceptions, reducing risk factors for secondary prevention, and what to expect during the hospital stay. Goals are set to reduce cardiovascular risk and increase physical activity. The patient is followed up via phone call to check in on goals General nurse led counseling involves providing generic advice, but no attempt at dispelling myths and misconceptions	Not specified, but measured using CDS	HeartOp Programme prior to surgery significantly reduced the severity of depressive symptoms, compared to usual care at 6 months postoperatively as measured by the CDS (Mean difference: 7.79, 95% CI: 2.04–13.54, *p* = 0.08)
Shuldham et al. (188)	To assess the benefit of preoperative education on improving postoperative pain, anxiety and depression	First time CABG	Randomized controlled trial	*n* = 269	Education group (*n* = 124) Control (*n* = 145)	Education was provided in a group setting (10–15 people) and covered pre-operative events and likely recovery process. The control group received informal one on one standard education. Questionnaires were sent, preoperatively, and at 6 weeks, 3 months and 6 months post-operatively	Not specified, but measured using HADS	There was no benefit from preoperative education (*n* = 124) on improving depressive symptoms measured by HADS compared to the usual care group (*n* = 145) at 3 days, 3 months and 6 months postoperatively (Mann–Whitney U: −11,886, Z: −0.50, *p* = 0.62)

CABG, coronary artery bypass graft; CDS, cardiac depression scale; HADS, hospital anxiety and depression scale.

Additionally, Furze et al. demonstrated that a nurse-led educational and cognitive behavioral intervention (HeartOp Programme) prior to surgery significantly reduced the severity of depressive symptoms, compared to usual care at 6 months postoperatively as measured by the CDS (Mean difference: 7.79, 95% CI: 2.04–13.54, *p* = 0.08) (187). Both groups initially received a 1-h interview in the outpatient clinic, followed by phone calls 1, 3, and 6 weeks later and then monthly communication until the procedure. In the HeartOp group, nursing staff addressed common cardiac misconceptions, discussed risk factor alterations for secondary prevention and expectations within the hospital setting. They also facilitated goal setting for risk factor reduction. The program appears to be economical, costing £288.83 per quality adjusted life year. Common myths and goal setting was not addressed in the usual care group.

Conversely, Shuldham et al. did not demonstrate a benefit from preoperative education (*n* = 124) on improving depressive symptoms measured by HADS compared to the usual care group (*n* = 145) at 3 days, 3 months and 6 months postoperatively (Mann–Whitney U: –11,886, Z: −0.50, *p* = 0.62) (188). Education was delivered through a 4-h group session with 10–15 patients and involved discussion of the pre/post-operative period and rehabilitation. In contrast, the usual care group received informal verbal advice at the time of admission. The lack of benefit is likely attributed to an optimal standard of preoperative education already provided by the hospital. On the other hand, shy and reserved patients may not have sufficiently engaged in group discussions despite having the opportunity. Future studies should also compare pre-operative education in a group setting compared to a regular patient consultation.

##### 4.1.1.2. Combination of pre-operative and post-operative education

In a Turkish population, Yaman Aktas et al. demonstrated preoperative and discharge education (*n* = 33) was effective in reducing HADS scores compared to usual care (*n* = 33) at 10 days and 4 weeks post-discharge following cardiac surgery (*F* = 19.23, *p* < 0.01) (191). Patients in the education group were provided one preoperative and four postoperative educational sessions. Preoperative education involved an informational pamphlet, an explanation of CHD, an overview of CABG and what to expect postoperatively. Postoperative education was commenced on postoperative day 3 to account for drowsiness and weakness immediately following surgery. Postoperative education involved an explanation of the recovery process, rehabilitation exercises, activities to avoid and when to re-present to hospital. The average education time per patient was 113.3 min, which appears justified given the reduction in depressive symptoms.

#### 4.1.2. Cardiac rehabilitation

Cardiac rehabilitation programs are focused on restoring a patient’s physical capacity following surgery, decreasing the likelihood of further cardiac events, and improving psychological wellbeing. Patients who develop postoperative depression while attending cardiac rehabilitation are commonly affected by comorbidities such as poor lifestyle choices, diabetes, chronic pain, or angina (192). These patients are more resistant to improvements in mental health. Future cardiac rehabilitation programs should aim to address these comorbidities.

Cardiac rehabilitation is effective in reducing depressive symptoms following cardiac surgery (Table 5) (193–195). The cardiac rehabilitation interventions have been variable in focus as well as timing. Hojskov et al. conducted early physical rehabilitation and psychoeducation following CABG over 4 weeks. The physical intervention consisted of deep breathing exercises, peak flow spirometry, walking, neck/shoulder and cycling exercises, whereas psychoeducation was provided over four face-to-face consultations with a focus on mindfulness (193). On the other hand, Ma et al. conducted a 12-month intervention involving not only physical exercise guidance, but also counseling on CAD-related health education, risk factor controls strategies and psychological monitoring (194). Cardiac rehabilitation has also been shown to be effective in reducing depressive symptoms even if the patient completes it from home (195). This significantly increases the accessibility of services, particularly for patients with transport or logistical difficulties when attending outpatient appointments.

**Table 5:** The effect of cardiac rehabilitation on improving depression following cardiac surgery.

Study	Aim	Surgery type	Study type	Patient number	Patient groups	Intervention	Definition of depression	Findings
Hojskov et al. (193)	Assess the effect of early cardiac rehabilitation (4 weeks following CABG) compared with usual care on 6 min walk time, anxiety and depression, physical and emotional scores and pain	CABG	Randomized controlled trial	*n* = 326	Early rehabilitation (*n* = 163). Usual care (*n* = 163)	Rehabilitation consisted of deep breathing exercises, peak flow spirometry, walking exercises, neck/shoulder exercises and cycling.	HADS ≥8	Early physical rehabilitation and psychoeducation following CABG decreased mean depression scores compared to usual care, as measured by HADS-D (Rehab: 3.7, Usual: 4.3) No effect on 6-min walk time
Ma et al. (194)	To assess the effect of a 12 month long comprehensive rehabilitation and intensive education program in patients with unprotected left main CAD who underwent CABG	CABG	Randomized controlled trial	*n* = 300	Rehabilitation program (*n* = 150) Usual care (*n* = 150)	Intervention consisted of CAD-related health education, physical exercise guidance, risk factor control strategies and psychological monitoring. Exercise guidance involved exercise selection and a prescription of frequency, intensity and duration of exercise. Conversely, usual care involved generic discharge education and information about their medications.	HADS ≥8 Evaluated at 3 months, 6 months and 12 months following admission	There was no significant difference in HADS-D score at baseline, 3 months, 6 months, and 9 months postoperatively. However, there was a reduction in HADS-D score at 12 months in the intervention group (mean value: −1.3±1.7) compared to the usual care group (mean value: −0.6±1.5, *p* < 0.001).
Takroni et al. (195)	To compare the effectiveness of home-based cardiac rehabilitation against outpatient based cardiac rehabilitation and usual care in a population of Saudi Arabian patients	CABG	Randomized controlled trial	*n* = 73	Home based cardiac rehab (*n* = 24) Outpatient based cardiac rehab (*n* = 25) Usual care (*n* = 24)	The outpatient cardiac rehabilitation group completed a physiotherapist-supervised program 3 times per week, with each session consisting of 15 min of warm-up, 20 min of progressive aerobic exercise and 10 min of cool-down. The home rehabilitation group completed a similar intervention, but selected exercises from an exercise library and received a weekly support call.	HADS ≥8 Evaluated at baseline at 8 weeks and 12 weeks.	Both the home-based and outpatient-based cardiac rehabilitation programs were effective in reducing HADS-D scores at 8 weeks (outpatient: 4.32, home: 4.88). However, the HADS-D score in the home-based rehabilitation program continued decreasing at the 4-week post-intervention follow-up (outpatient: 4.12, home: 3.79). High compliance rate in both the outpatient and home-based cardiac rehabilitation program at 91.7 and 87.5% respectively

CABG, coronary artery bypass graft; CAD, coronary artery disease; HADS, hospital anxiety and depression score.

### 4.2. Non-pharmacological management

#### 4.2.1. Cognitive behavioral therapy

Cognitive behavioral therapy (CBT) is effective, evidence-based psychotherapy for the management of psychopathologies such as depression and anxiety (196). CBT has several advantages. Firstly, the efficacy of CBT rivals that of anti-depressants in managing depression. Secondly, the concurrent use of CBT with antidepressants enhances the pharmacological effect of the medication and improves adherence to medications and rehabilitation plans (197, 198). Thirdly, the anti-depressive effect of CBT may last longer in comparison to anti-depressants and prevent relapse (199). Moreover, early initiation of CBT may halt the progression of new cases of depression. Persistent depressive symptoms are associated with a reduced chance of recovery. For example, the chance for recovery within the next 6 weeks if a patient has had depressive symptoms for 3 and 23 weeks is 40% and 5%, respectively, (200). CBT is limited by the necessity for patients to take ownership of their management, significant time commitments, travel requirements to clinic and limited focus on social networks (201, 202).

CBT sessions generally last an hour and run over several weeks. Within these sessions, trained mental health professionals will assist patients to identify automatic negative thoughts. They also aim to challenge and restructure negative thinking patterns and cognitive distortions with a goal to improve affect. In subsequent sessions, strategies aimed at maintaining psychological wellbeing are explored (203, 204). Following cardiac surgery, patients generally experience an improvement in psychological symptoms as their symptoms and quality of life significantly improve. However, postoperative pain, complications and slower than expected recovery may demotivate patients (205). This may also contribute to dysfunctional thoughts, loss of self-efficacy and fear of progression with rehabilitation exercises. CBT may directly address these cognitive distortions. Despite the clearly documented benefit of CBT in managing depression, there have been few studies exploring the efficacy of CBT in the cardiac surgery population (Table 6).

**Table 6:** Studies of cognitive behavioral therapy in improving depression following cardiac surgery.

Study	Aim	Surgery type	Study type	Patient number	Patient groups	Intervention	Definition of depression	Findings
Freedland et al. (206)	To compare the effect of CBT against supportive stress management and usual care on the management of depression following CABG	CABG	Randomized controlled trial	*n* = 123	CBT (*n* = 41) Supportive stress management (*n* = 42) Usual care (*n* = 40)	CBT sessions were hourly sessions once per week, over 12 weeks Supportive stress management aimed to improve the patient’s ability to cope with stressful life events through classes on breathing and relaxation techniques. Outcomes were measured at 3, 6, 9, and 12 months	HAM-D > 7 BDI ≥ 10	CBT (51%) was more effective than supportive stress management (33%) or usual care (15%) for inducing sustained remission of depression following CABG (χ^2^ = 11.95, *p* = 0.003). Improvements in major depression were only seen in the CBT group. In contrast, improvements in mild depressive symptomatology were observed in the CBT and supportive stress group—albeit the effect was greater in the CBT group. Supportive stress management was superior to usual care in reducing depressive symptoms at 3 months, but there was no difference at 6 or 9 months.
Dao et al. (207)	To assess the feasibility and accessibility of a brief tailored CBT intervention (MADES) on managing pre-operative depression	CABG	Randomized controlled trial	*n* = 97	MADES (*n* = 48) Usual care (*n* = 49)	The MADES group attended four sessions, including two preoperative and two postoperative sessions (within the week following cardiac surgery).	BDI ≥ 14	Compared to the usual care group, patients undergoing MADES had a shorter hospital stay (7.9 days ±2.6 days vs. 9.2 ± 3.5 days, *p* = 0.049). MADES was also associated with a reduction in depressive symptoms as confirmed by BDI, whereas patients receiving usual care demonstrated an increase in postoperative depressive symptoms. Low attrition rate (*n* = 1)
Doering et al. (208)	To assess the efficacy of early CBT conducted in the home environment by nurses in patients 1 month following CABG	CABG	Randomized controlled trial	*n* = 81	CBT (*n* = 45) Usual care (*n* = 36)	8 week CBT intervention	BDI ≥ 10	CBT group had a greater decrease in BDI scores compared to the usual care group. The BDI in the usual care group increased over the 8-week period (*β* = 1.41, 95% CI: 0.81–2.02). More CBT patients (*n* = 29, 64%) experienced remission from depression compared to the usual care group (*n* = 9, 25%).
Berensvaite et al. (69)	To assess the effect of CBT on HRV and its effectiveness on improving health related quality of life in patients 2 months following cardiac surgery	CABG	Randomized controlled trial	*n* = 89	CBT (*n* = 43) Usual care (*n* = 46)	9 month CBT intervention.	Not specified	At baseline (2 months postoperatively), the Mann–Whitney U test demonstrated the HRV was slightly lower in the CBT group compared to the usual care group (21.8 ms vs. 32.9 ms, U = 591.5, *p* = 0.047). At the final follow-up, there was a significant increase in the HRV within the CBT group compared to baseline (21.8 ms vs. 34.5 ms, *p* = 0.022). Conversely, the HRV decreased within the usual care group from baseline (32.9 ms vs. 29.2 ms, *p* > 0.05). Hence, CBT may improve the overall adaptability of autonomic nervous system. The LF/HF ratio did not significantly change for either group, suggesting that CBT did not alter overall autonomic balance.

BDI, beck depression inventory; CABG, coronary artery bypass graft; CBT, cognitive behavioral therapy; HAM-D, hamilton depression rating scale.

Standard delivery of CBT over a 12 week period appears to be effective at inducing sustained remission of depression following CABG compared to supportive stress management or usual care (χ^2^ = 11.95, *p* = 0.003) (206). To overcome the large time commitment required by traditional CBT models, a brief form of CBT such as the ‘Managing Anxiety and Depression Using Education and Skills’ (MADES) model have been proposed. For example, in the study by Dao et al., patients would attend 2 sessions preoperatively and 2 sessions within the first postoperative week. Compared to the usual care group, patients undergoing MADES had a shorter hospital stay (7.9 days ±2.6 days vs. 9.2 ± 3.5 days, *p* = 0.049) and demonstrated reduced depressive symptoms (207). However, the MADES model requires validation in a larger cardiac surgery population prior to widespread use. Furthermore, CBT has been shown to be effective in reducing depressive symptoms following cardiac surgery even when delivered at a patient’s home by nurses. This significantly eliminates accessibility and travel barriers required for patients to attend outpatient clinics (208). However, it should be noted that within this study, the number of females were under-represented within the CBT. This is a significant limitation as females have been shown to be more resistant to the effects of CBT (209). Lastly, a 9 month long CBT intervention following CABG has also been shown to be effective in improving the overall adaptability of the autonomic system. However, CBT did not improve vagal outflow (69).

#### 4.2.2. Religiousness, faith, and prayer

The lead up to cardiac surgery induces anxiety in many patients. Patients may cope using active or maladaptive coping strategies. Active coping strategies include behavioral or cognitive strategies. Behavioral coping strategies include actions to improve a situation, while cognitive strategies involve mental activities such as changing one’s perspective on the situation, and positive reappraisal (210). Maladaptive coping strategies include actions which do not attempt to improve the situation, but rather ignore it or make it worse.

Religion is an unrecognized psychosocial factor and coping mechanism which may affect recovery following cardiac surgery (211). Religiousness refers to the belief in religious doctrines or the involvement in religious practices such as attending services or prayer (212). Religious involvement is postulated to improve postoperative recovery. Attending religious services is associated with increased social support, leading to greater connection with congregation members. Secondly, regular attendance of religious services may influence cognitive appraisal processes, which may therefore modulate immunological, autonomic and neuroendocrine activity (213).

The effect of religion and prayer on health outcomes in patients following cardiac surgery is not well established (Table 7). Contrada et al. reported stronger religious beliefs were associated with reduced postoperative complications and a shorter length of hospital stay. However, when postoperative complications were controlled for, there was no relationship between religion and length of stay (211). Religious involvement was also associated with an improvement in depressive symptoms. However, attendance at religious events was associated with poorer recovery and increased length of hospital stay. This may be attributed to whether a patient has a positive or negative religious coping style. Negative religious coping styles are seen in patients with religious struggles, and who have an insecure relationship with their god. Individuals with spiritual conflicts report poorer wellbeing scores, including depression (216).

**Table 7:** Studies assessing the effect of religion on depressive features in patients undergoing cardiac surgery.

Study	Aim	Surgery type	Study type	Patient number	Patient groups	Intervention	Definition of depression	Findings
Contrada et al. (211)	To assess the effect of religiousness on depressive symptoms following cardiac surgery	CABG ± valve surgery	Prospective observational trial	*n* = 142	Single group	Religious and psychological factors measured 1 week prior to surgery	Not defined Measured using BDI	Stronger religious beliefs were associated with reduced postoperative complications and length of stay However, once postoperative complications were controlled for, there was no relationship between religion and length of stay. More frequent religious attendance is associated with poorer recovery and increased hospital stay Depressive symptoms are associated with longer hospital stay
Ai et al. (214)	To assess the role of private prayer and religiousness on depressive symptoms following cardiac surgery	CABG	Retrospective observational trial	*n* = 151	Single group	Questionnaires sent at 6 months and 1 year post cardiac surgery	Not defined Measured using Symptom Checklist-90R	Patients who prayed were less likely to be depressed at 1 year postoperatively, even when controlling for depression at 1 month post-operatively.
Ai et al. (215)	To assess the effect of private prayer as a coping mechanism and its relationship with optimism in patients undergoing cardiac surgery	CABG	Prospective observational trial	*n* = 226	Single group	First in person interview was conducted 2 weeks prior to surgery. Optimism was measured the day before surgery via telephone.	Not defined Measured using CES-D	Private prayer prior to CABG was associated with higher levels of hope and optimism before their surgery

BDI, beck depression inventory; CABG, coronary artery bypass graft; CES-D, center for epidemiological studies depression scale.

Ai and colleagues postulated that the use of prayer when faced with a medical issue draws allows an individual to draw upon one’s inner spirituality, and does not necessarily relate to specific religion or faith (214). Particularly in spiritual patients, the use of prayer may indicate a survival instinct and may also reduce anxiety (217). Patients who engaged in private prayer postoperatively had a lower level of distress 1 year following surgery (214). Ai et al. reported that private prayer prior to CABG was associated with higher levels of hope and optimism before their surgery (215). In contrast, Contrada and colleagues did not find an association between prayer and health outcomes or quality of life (211). They documented the frequency of prayer, but not the intent behind it. Clinicians should recognize that spirituality, religion, and prayer may be effective methods of coping. Spiritual guidance and services from a hospital chaplain or pastor should be offered to all patients.

#### 4.2.3. Postoperative education alone

Postoperative education has been uniquely delivered via an online format. Ma et al. compared an internet-based education and rehabilitation program (*n* = 70) against standard care (*n* = 70) for patients following CABG (218). They designed an educational rehabilitation program on the social media platform WeChat, the main communication modality in China (219). A 12-month intervention delivered by nurses involved health education, rehabilitation guidance, exercise supervision and psychological care. Health education involved the delivery of weekly videos on post-CABG management/recovery and modification of cardiovascular risk factors. Videos demonstrating rehabilitation exercises were created. Additionally, patients received fortnightly calls from nurses to discuss their psychological wellbeing. In comparison, the usual care group received once-off verbal advice prior to discharge and a call every month to discuss further rehabilitation guidance. At 12 months postoperatively, the HADS score was significantly lower in the WeChat group compared to the usual care group (5.2 ± 2.5 vs. 6.1 ± 3.1, *p* = 0.048). Internet-based rehabilitation programs are more convenient for patients and are not limited by travel requirements. These interventions are cost-effective and patient queries are promptly answered. It is important to monitor adherence to medication or rehabilitation, as internet-based education may result in reduced patient engagement.

#### 4.2.4. Biobehavioral interventions

Respiratory sinus arrythmia refers to the rhythmic increases and decreases in heart rate associated with respiration. Given respiratory sinus arrythmia is largely driven by vagal outflow and the pathophysiology of depression involves autonomic dysfunction, it reasonable to target this underlying mechanism. Patients in the biofeedback group may be trained to breathe abdominally and at a slower rate to synchronize their heart rate and abdominal breathing.

Despite limited data, Patron et al. found that biofeedback training to increase respiratory sinus arrythmia appears to be effective in decreasing depressive symptoms following cardiac surgery (220). However, given no follow up studies were conducted, it is unknown whether the improvements in depressive symptoms are long lasting. Additionally, it is also unclear whether depressive symptoms are reduced due to an improvement in autonomic regulation, or by another pathway. No other recent studies have been identified to examine the effect of biobehavioral interventions on depression following cardiac surgery.

### 4.3. Pharmacological management

#### 4.3.1. Antidepressants

The management of moderate to severe depression may involve psychological or pharmacotherapy. Psychotherapy has been shown to be equivalent pharmacotherapy in managing depression. Over 50% of patients do not respond to their initially prescribed antidepressant, and over 30% of patients do not respond to subsequent management (221). The most used antidepressants include selective serotonin reuptake inhibitors (SSRIs), serotonin and noradrenaline reuptake Inhibitors (SNRIs) and tricyclic antidepressants (222). For cardiac surgery patients, SSRIs and SNRIs are most commonly used (12). Examples of SSRIs include escitalopram, citalopram, fluoxetine, paroxetine and sertraline (222). Tricyclic antidepressants are largely avoided given their cardiotoxic nature (223).

The main aim of antidepressant therapy is to reduce psychological and physical symptoms and improve their functional capacity. Antidepressants should be used in conjunction with psychotherapies (224). SSRIs competitively inhibit the presynaptic uptake of serotonin, consequently increasing the level of serotonin within the brain (222). Moreover, they have also been reported to attenuate autonomic dysfunction through improvements via HRV and have anti-inflammatory effects (225). Recent evidence suggests that anti-depressants may exert cardioprotective properties the attenuation of platelet function by interfering with serotonin uptake within platelets. This may reduce the development and progression of atherosclerotic lesions, and thus reduce the risk of CHD (226).

##### 4.3.1.1. Efficacy

Despite limited evidence, SSRIs appear to be efficacious in reducing depression following cardiac surgery (Table 8) (227, 228). Prophylactic treatment with 10 mg of Paroxetine for 10 days in individuals identified to be at high risk of postoperative depression (older than 70 years and underwent emergency surgery) was associated with significantly lower CES-D scores (15.2 ± 7.8) compared to the control group (21.8 ± 7.5, *p* = 0.0018). The incidence of depression was significantly higher in the non-paroxetine group compared to the paroxetine group (64.4% vs. 12.1%, *p* < 0.0001) (227). Similarly, prophylactic treatment with 10 mg of escitalopram daily for 6 months was effective in reducing the mean BDI score from baseline compared to placebo in CABG patients (*p* = 0.015) (228). Moreover, the use of escitalopram in patients with pre-operative depression reported swifter improvements in quality of life and reduced postoperative pain. It should be noted that escitalopram was imitated starting 2–3 weeks prior to surgery to account for the delay period before the beneficial effects of SSRIs become clinically apparent (234).

**Table 8:** Studies assessing selective serotonin reuptake inhibitors in cardiac surgery patients.

Study	Aim	Surgery type	Study type	Patient number	Patient groups	Intervention	Definition of depression	Findings
Hata et al. (227)	To assess the efficacy of prophylactic management with selective serotonin reuptake inhibitors in females identified as high risk of developing depression postoperatively	Cardiac surgery	Case control	*n* = 117	Prophylactic SSRI (*n* = 58) No prophylactic SSRI (*n* = 59)	Intervention group received prophylactic treatment with 10 mg paroxetine in the first postoperative day, for 10 days. Patients in this group were also >70 years or undergone emergency surgery.	CES-D > 16	Depressive symptoms (mean ± SD) were measured using the CES-D at 10 days postoperatively. The group receiving paroxetine had significantly lower CES-D scores (15.2 ± 7.8) compared to the control group (21.8 ± 7.5, *p* = 0.0018). The incidence of depression was significantly higher in the non-paroxetine group compared to the paroxetine group (64.4% vs. 12.1%, *p* < 0.0001). The length of hospital stay was also significantly shorter in the prophylactic paroxetine group compared to the control group (15.9 days vs. 23.4 days, *p* = 0.0102).
Chocron et al. (228)	To assess the effect of prophylactic treatment with escitalopram on depression	CABG	Randomized controlled trial	*n* = 361	Escitalopram (*n* = 182) Placebo (*n* = 179)	Patients randomized (1:1) to escitalopram or placebo. Patients received 10 mg escitalopram 2–3 weeks prior to surgery, which continued until 6 months postoperatively.	BDI ≥ 4	10 mg of escitalopram daily reduced the mean BDI score more quickly from baseline at the six-month postoperative follow-up compared to placebo in CABG patients (*p* = 0.015). Moreover, the use of escitalopram in patients with pre-operative depression reported swifter improvements in quality of life and reduced postoperative pain. No differences in morbidity and mortality at 12 months.
Kim et al. (229)	To assess the safety of SSRIs in patients who have undergone CABG	CABG	Retrospective observational study	*n* = 1,380	SSRIs (*n* = 1,076) No SSRIs (*n* = 304)	Primary endpoint was defined as a composite of in hospital mortality, bleeding events	Not defined	No significant difference in the occurrence of the primary endpoint between the groups (9.4% vs. 8.2%, OR: 1.03, 95% CI: 0.60–1.78). Subgroup analysis of patients on antiplatelet and anticoagulation therapy did not reveal increased rate of bleeding. Patients on warfarin not included in subgroup analysis.
Tully et al. (230)	To assess the effect of SSRI/SNRIs on surgical morbidity and mortality following CABG	Primary isolated CABG	Prospective observational study	*n* = 4,136	SSRI/SNRI (*n* = 105) Non-SSRI/SNRI (*n* = 4,031)	Morbidity and mortality outcomes were recorded from electronic database of patients undergoing cardiothoracic surgery between 1996 and 2008	Not defined	Median follow up time was 4.7 years (interquartile range 2.3–7.9 years) Use of SSRI/SNRIs at the time of cardiac surgery did not increase all-cause mortality (HR: 1.03, 95% CI: 0.62–1.72, *p* = 0.91), cardiac mortality (HR: 0.31, 95% CI: 0.04–2.26, *p* = 0.25) or bleeding, even in patients receiving concomitant anti-platelet therapy (*p* > 0.20). There was an increased requirement for renal dialysis in patients receiving SSRI/SNRI (OR: 2.18, 95% CI: 1.06–4.45, *p* = 0.03) and an increased duration of mechanical ventilation postoperatively (OR: 1.69, 95% CI: 1.03–2.78, *p* = 0.04).
Heimisdottir et al. (231)	To assess the effect of SSRI/SNRIs on bleeding following CABG	Primary isolated CABG	Retrospective observational study	*n* = 1,237	SSRI/SNRIs preoperatively (*n* = 97) Reference group (*n* = 1,140)	Bleeding assessed using 24 h chest tube output, number of packed red blood cells transfused and reoperation for bleeding	Not defined	There was no significant difference in the mean 24-h chest drain output (SSRI/SNRI: 815 mL vs. Control: 877 mL, *p* = 0.26), the number of packed red blood cell transfusions (SSRI/SNRI: 2.2 vs. Control: 2.2, *p* = 0.99) and the re-operative rate (SSRI/SNRI: 4.1% vs. Control: 6.0%, *p* = 0.61).
Xiong et al. (232)	To assess the long term outcomes resulting from SSRI use prior to CABG	CABG	Prospective observational study	*n* = 4,794	SSRIs preoperatively (*n* = 246) No SSRIs (*n* = 4,548)	Morbidity and mortality data was collected between 1999 and 2003	Not defined	Median follow up time: 3 years Use of SSRIs before CABG was associated with a higher risk of long-term mortality and rehospitalization following surgery (HR: 1.52, 95% CI: 1.30–1.77, *p* < 0.0001)
Stenman et al. (233)	To assess the association between preoperative antidepressant use and survival following CABG	Primary, isolated non-emergent CABG	Retrospective observational study	*n* = 10,884	Anti-depressants pre-operatively (*n* = 1,171) No pre-operative antidepressants (*n* = 9,713)	Morbidity and mortality data collected between 2006 and 2008 using the SWEDHEART registry	Not defined	Preoperative antidepressant use was associated with increased mortality compared to patients without anti-depressants (Adjusted HR: 1.45, 95% CI: 1.18–1.77) following adjustment for diabetes, COPD and left ventricular dysfunction Antidepressant use was also associated with an increased risk of rehospitalization (HR 1.40; 95% CI 1.19–1.65)

BDI, beck depression inventory; CABG, coronary artery bypass graft; CES-D, center for epidemiological studies of depression scale; HR: hazards ratio, SNRI, serotonin and norepinephrine reuptake inhibitors; SSRI, selective serotonin reuptake inhibitor.

##### 4.3.1.2. Safety profile

Studies of the safety profile of antidepressants in the cardiac surgery population have mainly involved assessment of morbidity, mortality, and the risk of bleeding.

***Mortality:*** The evidence describing the effect of SSRI/SNRIs on mortality in patients following cardiac surgery has been conflicting. Tully et al. reported that use of SSRI/SNRIs at the time of cardiac surgery did not increase all-cause mortality (HR: 1.03, 95% CI: 0.62–1.72, *p* = 0.91) or cardiac mortality (HR: 0.31, 95% CI: 0.04–2.26, *p* = 0.25) (230). Chocron et al. also did not report a difference in mortality between patients who were taking 10 mg of escitalopram and the control group (228). Similarly, Kim et al. reported no significant difference in the composite endpoint of hospital mortality and bleeding in patients who used antidepressants (9.4% vs. 8.2%, OR: 1.03, 95% CI: 0.60–1.78) (229). A systematic review comprising 162,001 patients (with 9,751 using SSRIs) revealed that the use of SSRIs pre-operatively or postoperatively did not increase 30-day hospital mortality or long-term mortality (235).

Conversely, Xiong et al. reported that use of SSRIs before CABG was associated with a higher risk of long-term mortality and rehospitalization following surgery (HR: 1.52, 95% CI: 1.30–1.77, *p* < 0.0001) (232). Notably, patients in the SSRI group were more likely to be affected by diabetes, dyslipidemia, hypertension, cerebrovascular disease, peripheral vascular disease, and a family history of coronary artery disease. These metabolic syndrome risk factors may have contributed to the poorer health of the patients in the SSRI group. Additionally, Xiong and colleagues did not adjust for potential covariates such as renal dysfunction and left ventricular dysfunction, factors which may significantly contribute to the increased mortality. Moreover, they only reported 5.1% of patients used SSRIs prior to surgery, while the prevalence of preoperative depression in CABG is estimated to be approximately 20% (12). This means patients may be underdiagnosed and undertreated, which inherently lead to poorer outcomes. Additionally, Stenman et al. also observed that preoperative antidepressant use was associated with increased mortality (HR: 1.45, 95% CI: 1.18–1.77) following adjustment for diabetes, COPD and ventricular dysfunction (233). This is concerning and warrants further investigation into the safety of SSRIs in the cardiac surgery population.

***Bleeding:*** Concerns have been raised regarding the potential bleeding risk of SSRIs/SNRIs. Bleeding risk is particularly heightened in a cardiothoracic surgery population given they are at increased risk of rhythm disorders and are likely already on anti-platelet or anti-coagulants. SSRIs do not appear to increase bleeding risk in the vulnerable cardiac surgery population. Kim et al. performed a sub-analysis within their study which demonstrated no increased bleeding risk in patients already on anti-platelets or direct oral anticoagulants (229). However, patients on warfarin were not included in this sub-analysis, warranting future investigations into this common cardiac surgery demographic. Similarly, Tully et al. did not report increased bleeding risk from SSRIs, even in patients receiving anti-platelet therapy (*p* > 0.20) (230). Lastly, Heimisdottir et al. demonstrated that there was no significant difference in the mean 24-h chest drain output (SSRI/SNRI: 815 mL vs. Control: 877 mL, *p* = 0.26), the number of packed red blood cell transfusions (SSRI/SNRI: 2.2 vs. Control: 2.2, *p* = 0.99) and the re-operative rate (SSRI/SNRI: 4.1% vs. Control: 6.0%, *p* = 0.61) (231).

***Other adverse reactions:*** The rate of adverse drug reactions to SSRIs/SNRIs appears to be low. Some examples of more common adverse reactions include diarrhea, nausea, vomiting, constipation, shivering and peripheral neuropathy (228). Notably, Tully and colleagues observed an increased requirement for renal dialysis in patients receiving SSRI/SNRI (OR: 2.18, 95% CI: 1.06–4.45, *p* = 0.03) and an increased duration of mechanical ventilation postoperatively (OR: 1.69, 95% CI: 1.03–2.78, *p* = 0.04) (230). However, these are novel results and should be interpreted with caution. SSRIs, especially citalopram, have also been associated with QTc prolongation. Rarely, they are associated with ventricular arrythmias and Torsade’s- de-Pointes. Consequently, the United States Food and Drug Administration do not recommend dosing citalopram higher than 40 mg/day (223).

#### 4.3.2. Statins

Statins are predominantly used to manage hypercholesterolemia, a modifiable risk factor which strongly contributes to CHD (236). Mechanistically, statins reduce cholesterol biosynthesis within hepatocytes through competitive inhibition of 3-hydroxy-3-methyl glutaryl- coenzyme A (HMG-CoA) reductase (237). This results in an increased hepatic uptake of cholesterol from the bloodstream, reduced concentration of total cholesterol, and slightly increased concentration of high-density lipoprotein (HDL). Statins have also been associated with a myriad of pleiotropic effects such as anti-inflammatory, anti-sclerotic, antioxidant and anti-depressant activity (238).

The brain is a metabolically demanding and lipophilic organ which is highly susceptible to reactive oxygen species and oxidative stress (239). Statins, particularly atorvastatin, can act as an antioxidant to counteract the imbalance of reactive oxygen species, preventing further neuronal damage (240). Additionally, statins have been demonstrated to regulate glutamate excitotoxicity, another potential contributor to the pathophysiological mechanism of depression (240). Interestingly, the antidepressant effects of statins are also dependent on serotonergic modulation. However, this mechanism is unclear.

Statins may be classified as lipophilic or hydrophilic. Examples of lipophilic statins include atorvastatin, simvastatin and fluvastatin. On the contrary, examples of hydrophilic statins include pravastatin and rosuvastatin (241). Generally, lipophilic statins readily penetrate the blood brain barrier (BBB). Notably, simvastatin can pass through the BBB at least six times more easily compared to atorvastatin. Simvastatin’s stronger ability to penetrate the BBB may explain the result of studies where simvastatin was found to protect against the onset of Alzheimer’s Dementia compared to other statins such as lovastatin (242). Statins are well tolerated and a safe drug. The common adverse drug reactions (over 1% incidence) include myalgia, gastrointestinal symptoms, sleep disturbances and transient elevations in liver function tests. More significant drug reactions include rhabdomyolysis (particularly if taken with other cytochrome p450 inhibitors), hepatic dysfunction and peripheral neuropathy. There are few drug–drug interactions with statins (222).

##### 4.3.2.1. Anti-inflammatory effect of statins

As previously discussed, depression may result in a pro-inflammatory state, which contributes to CHD. In turn, CHD and cardiac surgery itself is associated with a potentially a deleterious systemic inflammatory response which may contribute to neuroinflammation (101). Studies have demonstrated the anti-inflammatory properties of statins, even in the cardiac surgery population. Statins may indirectly attenuate the pro-inflammatory state through the reduction in LDL cholesterol (243). Chello et al. reported administration of atorvastatin (20 mg daily, n = 20) for 3 weeks in patients undergoing on-pump CABG resulted in significantly lower levels of IL-6 and IL-8 at four, and 24 h postoperatively (*p* = 0.02) compared to placebo (*n* = 20). They also observed a reduction in neutrophil CD18/CD11b expression at 4 h (*p* = 0.004) and 24 h (*p* = 0.01) postoperatively in the atorvastatin group compared to placebo. Chello and colleagues correlated this reduction in pro-inflammatory activity to reduced neutrophil adhesion in the saphenous vein endothelium (244). Similarly, a 2003 study by Chello and colleagues demonstrated an attenuation in neutrophil CD11b and endothelial P-selectin expression in the simvastatin group, compared with non-responders to simvastatin and the control group following on-pump CABG. This study supports the anti-inflammatory role of statins through a nitric oxide-mediated mechanism. Patients received statin therapy for a minimum of 3 months within this study to maximize the bioavailability of nitric oxide, but the average duration of treatment was not specified (245).

Moreover, the use of statins is associated with a reduction in CRP levels (246, 247). In a study comparing the effect of varying doses of atorvastatin (20 mg daily for 5 days, 80 mg for 4 days followed by 40 mg/day for a total of 5 days or no atorvastatin), the highest dose of atorvastatin was associated with a significant reduction in the CRP level (mean ± standard error of the mean). The CRP level in group B was 13,545 ± 959.9 mg/L.h (95% CIL 11,476 mg/L.h – 15,604 mg/L.h), whereas the CRP level in group A was 17,085 ± 858.4 mg/L.h (*p* = 0.01) (248).

##### 4.3.2.2. Efficacy of statins in preventing or reducing depression

Statins should only be used as adjuncts for depression when further evidence regarding their efficacy emerges. From preliminary evidence, statins appear to prevent and improve depressive symptoms in the cardiac surgery population (Table 9). Stafford and Berk demonstrated the use of statin therapy commenced upon discharge was associated with a 79% reduction in the likelihood of developing MDD at 9 months post-operatively (95% CI: 0.052–0.876, *p* = 0.032) (249). Patients were started on either atorvastatin (*n* = 114), simvastatin (*n* = 29) or pravastatin (*n* = 14). The study was limited by an unclear adherence rate to the statins at 9 months. Hence, it is unclear how much the statins contributed to the reduced likelihood of depression. Notably, the patient cohort also consisted of patients who were hospitalized for CABG and percutaneous transluminal angioplasty or myocardial infarction. Despite the inclusion of three different presentations, the authors treated these patients as one homogenous group given evidence suggests CHD is both an etiological and prognostic factor for depression (251). Additionally, Abbasi et al. demonstrated that after 6 weeks, patients receiving simvastatin (20 mg daily) experienced a reduction in depressive symptoms (*p* = 0.026) (250). While the average Hamilton Depression Rating Scale score was lower in the simvastatin group (4.95 ± 3.98) compared to the atorvastatin group (8.56 ± 6.50), there was no significant difference between the groups. Future studies should have a larger sample size to compare simvastatin against placebo, as well confirm the potential superiority of simvastatin to other statins in preventing or managing depression following cardiac surgery.

**Table 9:** Studies assessing the efficacy of statins in preventing or reducing depression in cardiac surgery patients.

Study	Aim	Surgery type	Study type	Patient number	Patient groups	Intervention	Definition of depression	Findings
Stafford and Berk (249)	To determine whether statins are associated with reduced risk of depression in patients who have undergone a cardiac intervention	CABG or angioplasty	Prospective observational study	*n* = 193	Receiving statins at discharge (*n* = 157) Not receiving statins (*n* = 36)	Patients followed up for total of 9 months. Assessed at 3 months and 9 months using the Mini international neuropsychiatric interview and HADS	HADS ≥8	Use of statin therapy at discharge was associated with a 79% reduction in the likelihood of developing MDD at 9 months post-operatively (95% CI: 0.052–0.876, *p* = 0.032)
Abbasi et al. (250)	To compare the effect of simvastatin and atorvastatin on improving mild to moderate depression in patients who recently underwent CABG in the previous 6 months	CABG	Randomized, double blind, placebo-controlled trial	*n* = 46	Simvastatin (*n* = 23) Atorvastatin (*n* = 23)	6 weeks of treatment with either simvastatin (20 mg daily) or atorvastatin (20 mg daily) Patients were followed up at baseline, 3 weeks and 6 weeks	Hamilton depression rating scale used Score for depression not defined	There was a significant reduction in depressive symptoms, as quantified by the HDRS compared to baseline in the simvastatin group (*p* = 0.026) at the final week of the study. However, there was no significant reduction in depressive symptoms upon comparison between groups. Moreover, Kaplan Meier estimation demonstrated a quicker reduction in depressive symptoms in the simvastatin group compared to atorvastatin group (*p* = 0.026)

CABG, coronary artery bypass graft; HADS, hamilton anxiety and depression scale; HDRS, hamilton depression rating scale; MDD, major depressive disorder.

### 4.4. Alternative models of care

Collaborative Care is an emerging model of healthcare whereby a health professional, most commonly a nurse, acts as a case manager and facilitates communication between the patient and multi-disciplinary team. Based on Wagner’s Chronic Care Model, the case manager acts under the supervision of a primary care physician to educate patients about their medical condition, actively listens to their concerns and treatment preferences, offers evidence-based management advice, liaises with the multi-disciplinary team and proactively monitors a patients’ response to treatment (252).

The Bypassing the Blues (BtB) trial was an 8 month, RCT funded by the National Institute of Health (US) to assess the potential role of collaborative care in the management of depression following cardiac surgery (253, 254). Patients were allocated to either the usual care group (*n* = 152) or collaborative care group (*n* = 150). Collaborative care patients were contacted by case managers to receive psychoeducation and discuss preferred treatment options. Subsequently, treatment plans were formulated and reviewed by psychiatrists or family physicians. At the 8-month follow up, the proportion of patients with greater than a 50% reduction in depressive symptoms from baseline as measured by the Hamilton Depression Rating Scale were significantly higher in the collaborative care group (*n* = 75/150, 50%) compared to the usual care group (*n* = 46/152, 29.6%, *p* < 0.01). These results highlight the potential for collaborative care to facilitate the management of depression in cardiac surgery patients.

Post-hoc analysis of the BtB trial demonstrated that the benefits of collaborative care were not associated with adjustments in antidepressant medications through the 8-month period (*p* = 0.06) (255). Thus, the benefit of collaborative care is likely to be derived from additional time and rapport built with the care manager, rather than medication alterations. Through a 12-month cost-effectiveness study, collaborative care was associated with a median saving of $2068 US compared to the usual care group (256). However, this did not reach significance (*p* = 0.30). Given the potential healthcare savings and cost-effectiveness of collaborative care, primary care physicians should be involved in these emerging treatment approaches for managing depression following cardiac surgery.

## 5. Future directions

Additional validation studies should be conducted for the commonly used depression questionnaires within the cardiac surgery population. This would allow for the establishment of definitive cut-offs for depression within this population. Further studies should also focus on whether somatic symptoms following cardiac surgery significantly overestimate the severity of depression, and hence whether different questionnaires need to consider this issue. Additionally, given anxiety is highly comorbid with depression, these two mood states should be assessed concurrently rather than separately.

There are limited studies assessing the pathophysiological mechanisms leading to postoperative depression in cardiac surgery patients. Further research into autonomic dysregulation and the mechanism between postoperative depression and cardiac surgery should be explored. Studies assessing HRV and autonomic dysregulation should control for respiratory rate and sinus arrhythmias, given they may affect HRV readings (257). HRV is not routinely collected in clinical practice. Institutions should consider using HRV biofeedback tools to track HRV and aim to normalize vagal outflow. Elevated levels of CRP are associated with postoperative depression and predicts the length of hospital stay. Studies should also assess the levels of other inflammatory markers such as IL-6, TNF-α and their association with postoperative depression. However, little is known about how this inflammatory state translates into postoperative depression. There should be increased examination of HPA dysregulation and the association with postoperative depression in cardiac surgery patients. Metabolic and genomic studies into this area may provide insight into the underlying mechanisms of HPA dysregulation and the association with depression. Studies should assess the concentration of CRH, ACTH and cortisol specifically in cardiac surgery patients to gain a holistic view of HPA axis functioning.

Future studies should assess the ideal duration and format of CBT (i.e., clinic vs. telephone based) to manage depression postoperatively. Given the costs associated with CBT, studies should attempt to identify which postoperative patients should undergo CBT. Very little is known about the mechanism of statins in reducing depressive symptoms, and thus, studies should also compare one statin at a time. For example, one statin should be compared to placebo, or another type of statin. Studies involving pharmacological agents should also include adherence rates, and how long patients were taking the medications for.

## 6. Conclusion

The understanding of the bidirectional relationship between depression and cardiac disease is limited. Depression is associated with several pathophysiological and behavioral factors which increase the likelihood of developing CHD. In addition, these factors may also contribute to postoperative depression. Additional studies are needed to elucidate these possible mechanisms, particularly within the cardiac surgery population who are highly susceptible to postoperative depression and a better understanding of such will further inform future prevention and management strategies.

## Author contributions

TV and JS conceptualized and designed the study. TV collected the data, analyzed the data, and produced the first draft of the manuscript. All authors contributed to subsequent drafts of the manuscript, including editing, and refining of the final manuscript and approved the final version of the manuscript for submission.

## Figures and Tables

**Table:** 

ADL	Activities of Daily Living
ADR	Adverse drug reactions
BBB	Blood Brain Barrier
BDI	Beck Depression Inventory
BtB	Bypassing the Blues
CABG	Coronary Artery Bypass Grafting
CBT	Cognitive Behavioral Therapy
CDS	Cardiac Depression Scale
CES-D	Centre for Epidemiological Study of Depression
CRH	Corticotrophin Releasing Hormone
CRP	C-Reactive Protein
CVD	Cardiovascular Disease
DSM	Diagnostic Statistical Manual of Mental Disorders
HADS-D	Hospital Anxiety, Depression Score – Depression subset
HDL	High Density Lipoprotein
HMG-CoA	3-Hydroxy-3-Methyl Glutaryl-Coenzyme A
HPA	Hypothalamic Pituitary Adrenal
HRV	Heart Rate Variability
IDO	Indolamine 2,3-Dioxygenase
IFN-γ	Interferon γ
IL-6	Interleukin-6
IL-8	Interleukin-8
LDL	Low-Density Lipoprotein
LF	Low Frequency
MACE	Major Adverse Cardiovascular Event
MADES	Managing Anxiety and Depression with Education and Skills
NMDA	N-methyl-D-aspartate
NYHA	New York Heart Association
PHQ	Patient Health Questionnaire
PTSD	Post Traumatic Stress Disorder
RCT	Randomized Controlled Trial
rMSSD	Root of Mean Square Difference in the N-N Intervals
TAVR	Transcatheter Aortic Valve Replacement
TDO	Tryptophan 2,3-Dioxygenase
TNF-α	Tumor Necrosis Factor α
SAVR	Surgical Aortic Valve Replacement
SNRI	Serotonin and Noradrenaline Reuptake Inhibitors
SSRI	Selective Serotonin Reuptake Inhibitors
SDNN	Standard deviation of mean R-R interval
VLF	Very Low Frequency
